# Antibody array-based proteome approach reveals proteins involved in grape seed development

**DOI:** 10.1093/plphys/kiad682

**Published:** 2024-02-23

**Authors:** Ying Zhang, Yiming Wang, Ruitao Liu, Zhangjun Fei, Xiucai Fan, Jianfu Jiang, Lei Sun, Xun Meng, Chonghuai Liu

**Affiliations:** National Key Laboratory for Germplasm Innovation & Utilization of Horticultural Crops, Zhengzhou Fruit Research Institute, Chinese Academy of Agriculture Sciences, Zhengzhou 450009, China; Chuxiong Yunguo Agriculture Technology Research Institute (Yunnan), Zhongyuan Research Center, Chinese Academy of Agricultural Sciences, Henan 450008, China; The Key Laboratory of Plant Immunity, Collage of Plant Protection, Nanjing Agricultural University, Nanjing 210095, China; National Key Laboratory for Germplasm Innovation & Utilization of Horticultural Crops, Zhengzhou Fruit Research Institute, Chinese Academy of Agriculture Sciences, Zhengzhou 450009, China; Boyce Thompson Institute for Plant Research, 533 Tower Road, Ithaca, NY 14853-1801, USA; National Key Laboratory for Germplasm Innovation & Utilization of Horticultural Crops, Zhengzhou Fruit Research Institute, Chinese Academy of Agriculture Sciences, Zhengzhou 450009, China; National Key Laboratory for Germplasm Innovation & Utilization of Horticultural Crops, Zhengzhou Fruit Research Institute, Chinese Academy of Agriculture Sciences, Zhengzhou 450009, China; National Key Laboratory for Germplasm Innovation & Utilization of Horticultural Crops, Zhengzhou Fruit Research Institute, Chinese Academy of Agriculture Sciences, Zhengzhou 450009, China; School of Life Science, Northwest University, Xi’an, Shanxi 710069, China; Abmart, 333 Guiping Road, Shanghai 200033, China; National Key Laboratory for Germplasm Innovation & Utilization of Horticultural Crops, Zhengzhou Fruit Research Institute, Chinese Academy of Agriculture Sciences, Zhengzhou 450009, China

## Abstract

Grape (*Vitis vinifera*) is one of the most widely cultivated fruits globally, primarily used for processing and fresh consumption. Seedless grapes are favored by consumers for their convenience, making the study of seedlessness a subject of great interest to scientists. To identify regulators involved in this process in grape, a monoclonal antibody (mAb)-array-based proteomics approach, which contains 21,120 mAbs, was employed for screening proteins/antigens differentially accumulated in grape during development. Differences in antigen signals were detected between seeded and seedless grapes revealing the differential accumulation of 2,587 proteins. After immunoblotting validation, 71 antigens were further immunoprecipitated and identified by mass spectrometry (MS). An in planta protein–protein interaction (PPI) network of those differentially accumulated proteins was established using mAb antibody by immunoprecipitation (IP)–MS, which reveals the alteration of pathways related to carbon metabolism and glycolysis. To validate our result, a seedless-related protein, DUF642 domain-containing protein (VvDUF642), which is functionally uncharacterized in grapes, was ectopically overexpressed in tomato (*Solanum lycopersicum* “MicroTom”) and led to a reduction in seed production. PPI network indicated that VvDUF642 interacts with pectin acetylesterase (VvPAE) in grapes, which was validated by BiFC and Co-IP. As anticipated, overexpression of *VvPAE* substantially reduced seed production in tomato. Moreover, *S. lycopersicum colourless non-ripening* expression was altered in *VvDUF642*- and *VvPAE*-overexpressing plants. Taken together, we provided a high-throughput method for the identification of proteins involved in the seed formation process. Among those, VvDUF642 and VvPAE are potential targets for breeding seedless grapes and other important fruits in the future.

## Teaser

A proteomics approach, employing a 20K monoclonal antibody array, reveals that the interaction of two proteins affects cell wall formation, influencing seed coat development and playing a crucial role in seedlessness (stenospermocarpy) in grapes.

## Introduction

Seed formation is one of the most important biological features to produce new generations of plants and the most important trait for agricultural crop production (Sreenivasulu and Wobus 2013). The size of seeds affects not only the evolutionary fitness but also the grain yield of crops. Grape (*Vitis vinifera*) is one of the most important economic crops worldwide. Grape seeds are generally reduced or removed since they are inedible and affect processing. Therefore, consumer demand for seedless grapes continues to increase, and growers are constantly seeking such grape varieties. However, how the natural seedless process occurs in grapes is still largely unknown. The investigation of the seed reproduction process will facilitate an understanding of the molecular mechanism of seed formation that can be employed for improving seed production and quality.

Natural seedless grape formation is classified into two major processes: parthenocarpy and stenospermocarpy. Parthenocarpy arises when fruit automatically develops in the absence of fertilization (Joldersma and Liu 2018). Stenospermocarpy is the most common mechanism of seedlessness in grape varieties, where pollination and fertilization occur but the embryo subsequently aborts (Cai et al. 2021). The natural seed abortion process of seedless grapes takes place after successful pollination (Korkutal 2005; Keller 2015). Both egg cells and pollen are normally developed and could be successfully fertilized (Korkutal 2005; Keller 2015). The seed abortion process is initiated 3 to 5 d after pollination and leads to seedlessness of grapes (Korkutal 2005; Keller 2015). However, the molecular mechanisms of the abortion process are still unclear. Recently, seedlessness has been one of the most prized quality traits in grapes intended for direct consumption as fresh fruit and raisins. Therefore, investigation of the seed reproduction process will benefit our understanding of this process and help to improve seedlessness breeding.

During the last few decades, researchers have continued to study the mechanisms of seedless grapefruit formation with genetic methods, crossbreeding, grafting, or chemical treatments. Quantitative trait locus (QTL) analysis indicated that the *SEED DEVELOPMENT INHIBITOR* (*SDI*) locus in grape chromosome 18 is the major source of seedlessness for commercial grapevine production (Lahogue et al. 1998). Subsequently, Pellerone and colleagues developed the SSR marker VMC7F2, which was closely linked with the SDI locus. Transcriptome analysis was employed to understand differential gene expression between seeded and seedless grapes (Nwafor et al. 2014; Wang et al. 2016). Over 1,000 differentially expressed genes were identified in those works, providing abundant potential genomic resources involved in this process, including GA-responsive genes, MADS-box transcription factors, ethylene-responsive transcription factors, and WRKY transcription factors. However, it is still difficult to characterize genes directly involved in seed production processes with genetic evidence. Recently, by using genetic approaches, Royo and colleagues illustrated that an arginine-197-to-leucine substitution in the MADS-box gene *AGAMOUS-LIKE 11* (*AGL11*) in the *SDI* locus is the major cause of seedlessness in grapes (Royo et al. 2018). The substitution in *VvAGL11* suppresses embryo development as well as seed coat lignification (Royo et al. 2018), suggesting that these processes may be essential for seed development in grapes. Silencing of *SlALG11*, a homolog of *VvAGL11*, causes seedlessness in tomato (*Solanum lycopersicum*) (Ocarez and Mejía 2016). Recently, it was shown that overexpression of *VvHDZ28* (*V. vinifera homeodomain-leucine zipper protein 28*) results in seedless fruit in tomato (Li et al. 2021). Although much effort has been made during the last few decades, *VvAGL11* and *VvHDZ28* are the only genes known to be related to grape seed formation. Identification of *VvAGL11* plays an important role in the physiological process of seedless grapes. However, it still could not illustrate how seed abortion was initiated. Therefore, it is necessary to utilize new techniques to provide information for understanding the molecular mechanisms underlying seed abortion progress.

There is considerable demand among researchers for investigating protein expression, localization, and modification with antibody-based techniques. However, the generation of specific antibodies against various proteins is a costly and time-consuming process. The large-scale production of antibodies is difficult to reproduce, especially in nonmodel species. An antibody library that targets a complete proteome would be ideal for antibody-based investigations of specific organisms. The generation and application of a monoclonal antibody (mAb) library were reported by Fujita et al. (1982). *Drosophila melanogaster* nervous system proteins were used as antigens to generate 148 mAbs, which were then used in immunohistochemical assays (Fujita et al. 1982). Different mAb libraries containing 100 to 1,000 mAbs were subsequently generated for antigens from various sources, including human liver mitochondrial proteins (Gao et al. 2006), plasma membrane proteins from lung cancer patients (Wang et al. 2010; Guergova-Kuras et al. 2011), soluble proteins from bamboo (*Bambusoideae*) shoots (Wu et al. 2006), and proteins from Arabidopsis (*Arabidopsis thaliana*) flowers (Shi et al. 2017). Recently, Human Protein Atlas-based research led to a rapid and low-cost approach for generating more than 24,000 antibodies (Uhlen et al. 2015). Extensive supporting information was provided by immunoprecipitation (IP) with antigens of interest and mass spectrometric (MS) identification. Moreover, antibodies could also be employed to investigate protein dynamics, protein localization, and protein–protein interactions (PPI) during the seed development process with immunoblotting, immunohistochemical staining, coimmunoprecipitation (co-IP), and enzyme-linked immunosorbent assays. Therefore, the mAb-based protein array method provides an efficient strategy for high-throughput screening of protein dynamics genome-wide.

DUF642 domain-containing proteins (VvDUF642) are highly conserved in spermatophyte plants (Vázquez-Lobo et al. 2012). Proteomics analyses have revealed that DUF642 proteins are associated with the plant cell wall (Jamet et al. 2006; Calderan-Rodrigues et al. 2019), suggesting the putative role of DUF642 in cell wall formation/development. In Arabidopsis, two DUF642 proteins, encoded by *At4g32460* and *At5g11420*, showed direct interaction with PECTIN METHYL ESTERASE3 (PME3) (Zúñiga-Sánchez and and Gamboa-de Buen 2012). Overexpression of *At4g32460*enhances PME activity during the seed germination process, whereas the absence of the DUF642 encoded by *At4g32460* reduces PME activity and results in the reduction in silique development and seed formation in Arabidopsis (Zúñiga-Sánchez et al. 2014). Furthermore, another DUF642 encoded by *At3g08030* specifically interacts with cellulose in Arabidopsis (Borner et al. 2003). Moreover, overexpression of *VqDUF642* from Chinese grape (*Vitis quinquangularis*) accelerates plant growth and pectin methylesterase activity in tomato (Xie and Wang 2016). Taken together, these findings indicated that DUF642 family proteins may be essential for seed developmental processes. However, the role of DUF642 in seed development is largely unknown in grapes.

In this study, we performed a mAb-based proteomics approach to analyze the development process of grape seeds. A group of protein spots that significantly changed during the development process was detected by the mAb array. IP–MS identification showed that proteins related to carbon metabolism, glycolysis, biosynthesis of amino acids, and protein processing in the endoplasmic reticulum were differentially accumulated in the seeded and seedless grapes. Moreover, several proteins, including triosephosphate isomerase (TPI), the ras-related protein RABE1c, a Grip22-like protein, DUF642, and the 50S ribosomal protein L3, were detected in only the seedless grapes and validated by Western blot (WB) analysis, suggesting that these proteins may affect grape seed formation. Tomato plants overexpressing *VvDUF642* aborted seed production, indicating the negative role of *VvDUF642* in seed development. A protein interaction network was established by using a high-quality mAb, and the interaction between VvDUF642 and VvPAE was further confirmed. Overexpression of *VvPAE* also affects seed production in tomatoes. Interestingly, we also found that the expression of a ripening-related gene *S. lycopersicum colorless non-ripening* (*SlCNR*) was significantly different in *VvDUF642-* and *VvPAE*-overexpressing plants. Taken together, our results provided a high-throughput method for detecting proteins involved in the development process, which may be useful for future functional investigation and the breeding processes in grapes.

## Results

### Generation of a grape mAb library for high-throughput proteome analysis

**Figure 1.**
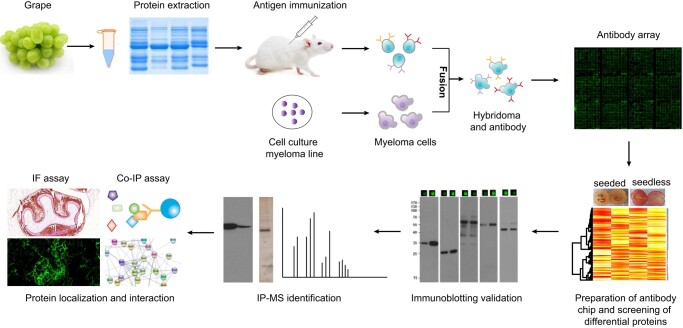
Schematic diagram of mAb library preparation. Proteins extracted from the embryo of seedless and seeded grapes at 15 d after anthesis were used to immunize mice to generate antigens. An mAb library was constructed after antibody clone screening and used for mAb array screening. The antibodies exhibiting fewer than three bands were considered effective antibodies and were used for IP, followed by mass spectrometric identification of targeting proteins.

The establishment of a large-scale mAb library is required for genome-wide high-throughput proteomic analysis of grape proteins (Wang et al. 2020). Significant alternation of genes involved in seed development was observed at 2 and 4 wk after anthesis (Royo et al. 2018). Therefore, an mAb library was generated by immunization of mice with proteins extracted from seeded and seedless grape berries at 15 and 35 days post-anthesis (DPA) and from leaves (Fig. 1, Supplementary Fig. S1). The development of grape seeds is classified into three major stages: the pro-embryo development stage (0 to 5 DPA), which is the initial stage that determines seed formation or abortion; the seed coat formation stage (normally 5 to 20 DPA); and the endosperm development formation stage (20 to 30 DPA). Here, we extracted proteins at 15 and 35 DPA, at which time there should be differences between proteins of well-developed and aborted grape seeds. This grape mAb library containing 21,120 qualified mAbs was successfully constructed with IgG > 0.5 mg ml^−1^ and used for the generation of a grape mAb array (Fig. 1). We randomly selected 312 antibodies to estimate the coverage of the grape mAb library. According to the WB analyses, 122 antibodies (approximately 39.1%) that exhibited less than three bands were considered effective mAbs that could be used for targeting protein identification (Supplementary Fig. S2). Accordingly, these results suggest that over 8,300 grape proteins could be used for target detection by using the 21,120 mAb library.

### Detection of proteins differentially expressed in seeded and seedless grapes with a mAb array

**Figure 2.**
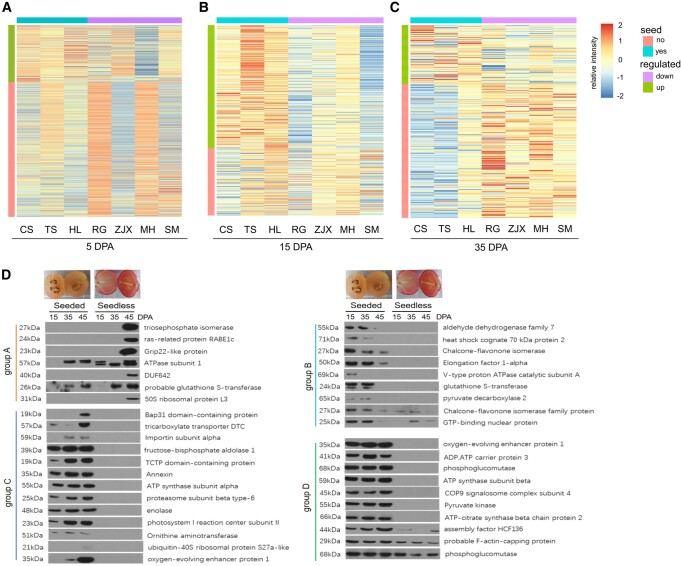
Detection of differentially expressed proteins during the seed development process by mAb library and WB analyses. **A to C)** Heatmap of differentially expressed proteins in the seedless grape cultivars (CS, TS, and HL), and seeded cultivars (RG, ZJX, MH, and SM) at 5, 15, and 35 DPA. **D)** WB analysis of identified proteins with a specific mAb in seeded (RG) and seedless (CS) grape fruits at 15, 35, and 45 DPA.

To understand the proteins involved in the seed development process, we performed mAb-based quantitative proteomics analysis by using proteins extracted from seeds or seed-like tissues of two different types of grapes. The first type is seedless grapes, including Centennial Seedless (CS), Thompson Seedless (TS), and Hong Lian (HL). The other type includes seeded grapes, Red Global (RG), Muscat Hamburg (MH), Shine Muscat (SM), and Zui Jinxiang (ZJX). Previous reports showed that ovule and embryo abortion was initiated at 5 to 10 d after full blooming (Joldersma and Liu 2018). Therefore, the seeds or seed-like tissues were collected at 5, 15, and 35 DPA, which represents the early, middle, and late stages of the seed formation process in grapes. Protein accumulation in the seed development process was screened by the grape mAb array. A total of 2,587 mAbs with significantly different signal intensities (*P* < 0.05) were identified (Fig. 2, A to C, Supplementary Table S1). Among those, 1,142 proteins were highly accumulated in seeded grapes, whereas 486 proteins were enriched in seedless grapes at 5 DPA (Fig. 2A). A total of 181 or 331 proteins and 237 or 105 proteins showed upregulation or downregulation at 15 and 35 DPA, respectively (Fig. 2, B and C).

To validate the mAb array results, WB analyses were further performed by using proteins extracted from seeded grapes (RG) and seedless grapes (CS) at 15 and 35 DPA, and the 45 DPA sample was also included to confirm subsequent protein changes. WB analyses with 39 randomly selected antibodies revealed different accumulations of those proteins in seeded and seedless grapes. These results association with the mAb array analysis results (Fig. 2D). However, this high-throughput mAb array only provides information on antigen signals, not on proteins. To identify differentially expressed proteins, IP coupled with MS identification was performed with these antibodies. A total of 71 proteins were identified based on the MS results (Supplementary Table S1). The identified proteins were further classified into four different groups based on their expression patterns. Group A included 7 proteins that were highly or specifically expressed in the seedless grapes, including TPI, the ras-related protein RABE1c, a Grip22-like protein, VvDUF642, and the 50S ribosomal protein L3 (Fig. 2D). Groups B, C, and D contained proteins that were highly accumulated in seeded grapes. Group B included 9 proteins that were depleted during the seed maturation process in the seeded groups. The accumulation of Group C, which contained 13 proteins, increased significantly during the seed maturation process. Group D proteins were highly accumulated in seeded grapes, and their accumulation was not significantly changed during the seed formation processes. The differential expression pattern suggests that these proteins function slightly differently in different developmental stages.

**Figure 3.**
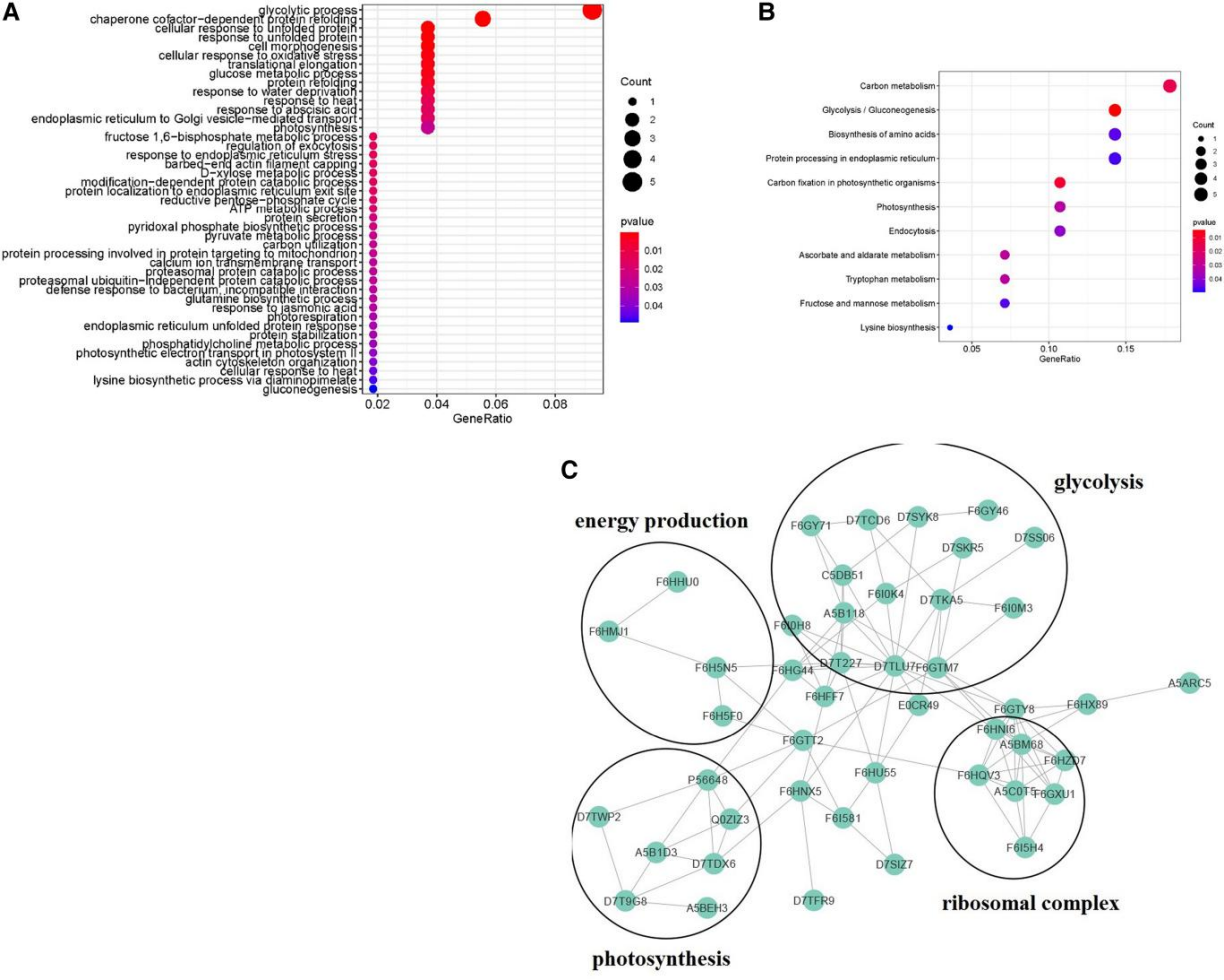
KEGG and regulatory network analyses of differentially expressed proteins during the seed development process in seeded and seedless grapes. KEGG analysis (**A, B)** and protein regulation network analysis (**C)** were performed by using differentially expressed proteins identified by IP–MS analysis.

Based on these results, we further performed KEGG enrichment analyses to identify the pathways that play a major role in the seed developmental process. KEGG enrichment analysis of identified proteins that were differentially expressed in the seeded and seedless grapes showed that the proteins were related to the glycolytic process, glutamine biosynthetic process, and glucose metabolic process in the biological process category (Fig. 3A). Moreover, in the molecular function category, the proteins were involved in carbon metabolism, carbon fixation in photosynthetic organisms, and glyoxylate and decarboxylate metabolism processes (Fig. 3B). Moreover, network analysis showed that the major proteins modified during the seed abortion process were closely related to glycolysis, energy production, photosynthesis, and ribosomal complexes (Fig. 3C). Taken together, these findings indicated that the regulation of energy production and carbon metabolism may be essential for seed developmental processes.

### Establish of PPI network during grape seed development

With the advantage of a high-quality mAb library, we established the PPI network of differentially expressed proteins by IP–MS. We randomly selected nine antibodies against proteins highly enriched in the seed grapes, and all seven antibodies against proteins highly/specifically accumulated in the seedless grapes. The following proteins interacted with proteins highly accumulated in seeded grapes: importin subunit alpha, annexin, fructose-bisphosphate aldolase1, AT synthase subunit alpha, proteasome subunit beta type-6, enolase, mitochondrial dicarboxylate/tricarboxylate transporter, oxygen-evolving enhancer protein1, and ubiquitin-40S ribosomal protein S27a-like (Supplementary Fig. S3A). Gene ontology (GO) enrichment analysis showed that these interacting proteins were related to carbon metabolism, glycolysis, and carbon fixation processes (Supplementary Fig. S3C). Similarly, the proteins that interacted with proteins highly accumulated in seedless grapes (Supplementary Fig. S3B) were also involved in carbon metabolism, biosynthesis of amino acids, and carbon fixation (Supplementary Fig. S3D). These results suggest that the regulation of carbon metabolism is closely related to the seed development process in grapes.

### Interaction network analysis of VvDUF642 in grape

**Figure 4.**
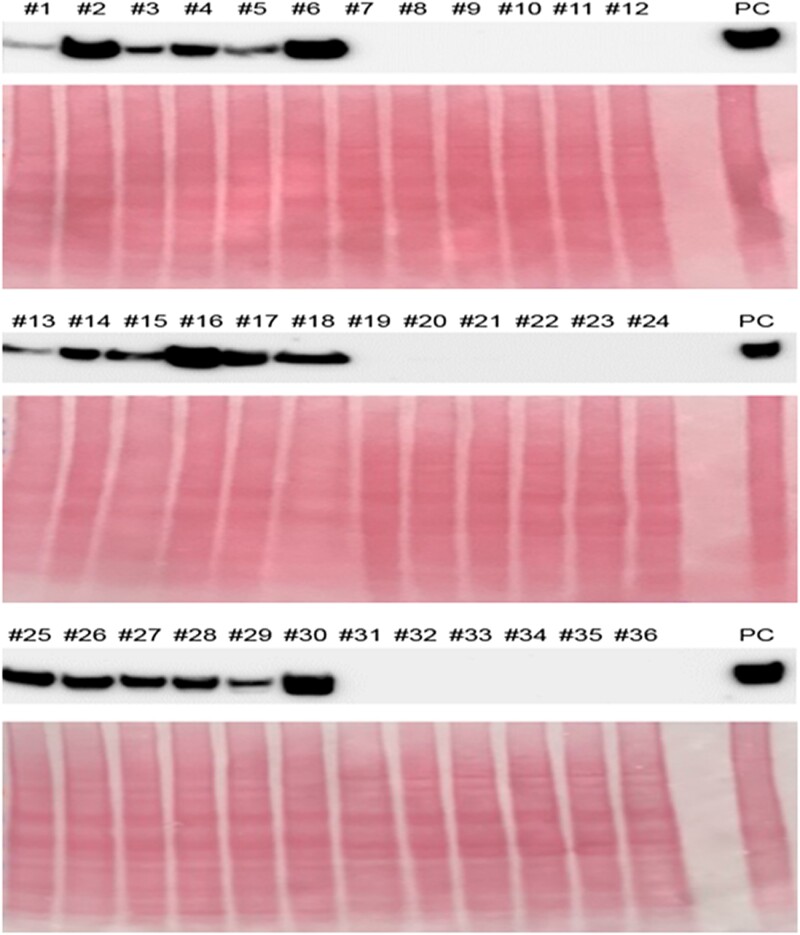
Protein accumulation of VvDUF642 in seeded and seedless grapes. Immunofluorescence of VvDUF642 in different grape cultivars. Grape cultivar: 1, Crimson seedless (seedless); 2, Hongwuzilu (seedless); 3, Honglian seedless (seedless); 4, Ruby seedless (seedless); 5, Zhengyan (seedless); 6, Feihong (seeded); 7, Yangputao (seeded); 8, Alexander (seeded); 9, Qianbao2 (seeded); 10, Cuixiangbao (seeded); 11, Zijixin (seeded); 12, Ziputao (seeded); 13, Bixiang (seedless); 14, Hongguang (seedless); 15, Thompson seedless (seedless); 16, Qiuwuhe (seedless); 17, Meiliwuhe (seedless); 18, Liming seedless (seedless); 19, Zaomeigui (seeded); 20, Xiaobaiputao (seeded); 21, Xiehuahong (seeded); 22, Mascat Hamburg (seeded); 23, Zitao (seeded); 24, Zaoweila (seeded); 25, Qimiao seedless; 26, Dawuhezi (seedless); 27, Zaowuhebai (seedless); 28, Youwuhe (seedless); 29, Yanggeer (seedless); 30, Wuhezi (seedless); 31, Zhengzhouzaohong (seeded); 32, Yilixiangputao (seeded); 33, Zhengguo28 (seeded); 34, Zexiang (seeded); 35, Zeyu (seeded); 36, Zizhengzhu (seeded); Positive control (PC), Sentianni. Three independent experiments were performed with similar results.

Since DUF642 family proteins are highly related to cell wall modification and seed development in plants (Zúñiga-Sánchez and and Gamboa-de Buen 2012; Zúñiga-Sánchez et al. 2014; Xie and Wang 2016), we chose VvDUF642 for further validation. A natural grape cultivar collection, which contains 18 seeded and seedless grapes, respectively, was used to confirm the expression pattern of VvDUF642. Total proteins were extracted from grapes at 45 DPA. VvDUF642 was specifically detected in all 18 seedless grape cultivars, but not in the seeded grapes (Fig. 4). These results indicate VvDUF642 may be associated with the seed abortion process.

**Figure 5.**
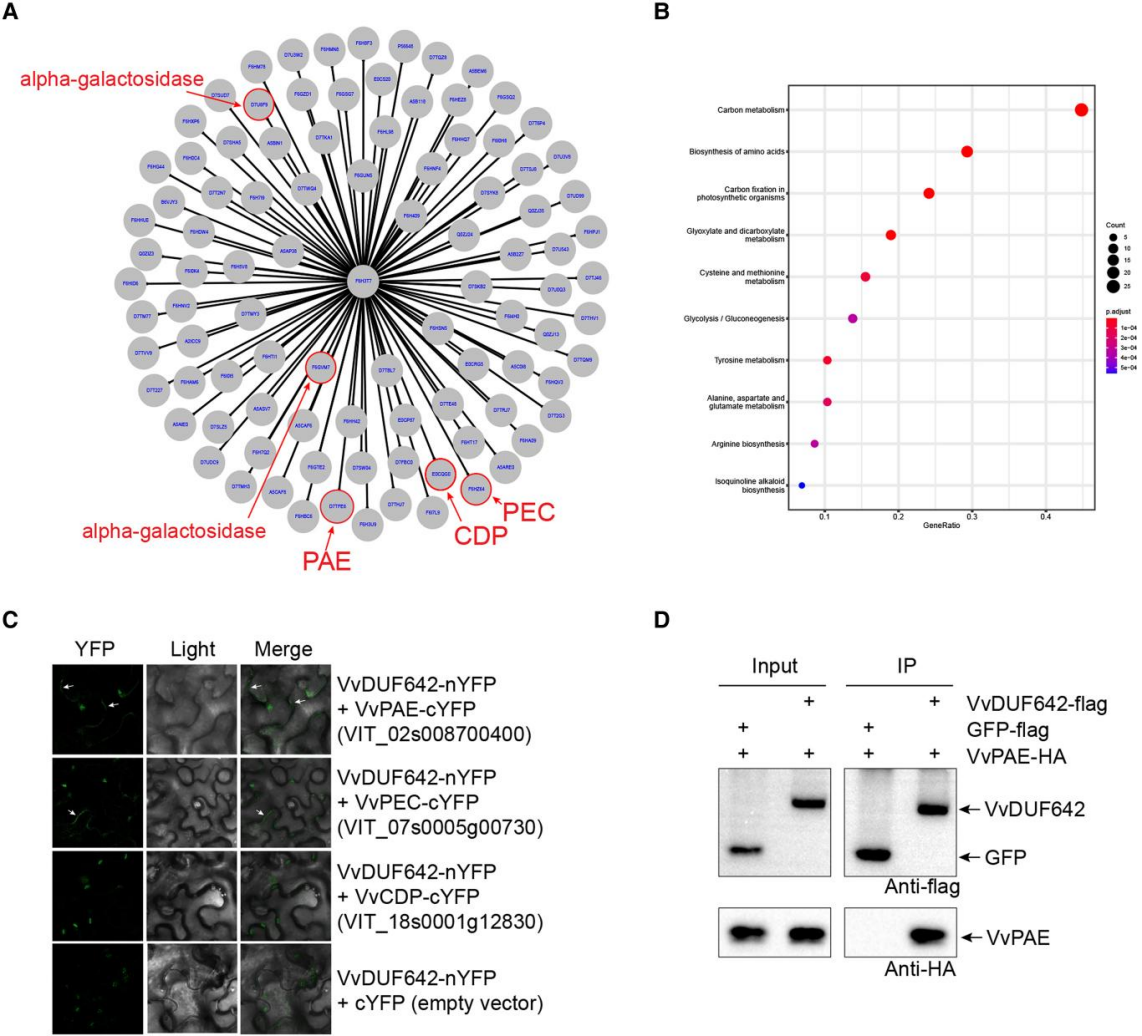
PPI network and interaction validation of VvDUF642. **A, B)** PPI network (**A**) and KEGG analysis (**B**) of VvDUF642 was generated by interacting proteins identified by IP–MS. The circles indicate proteins related to cell wall modifications. **C)** BiFC analysis of VvDUF642 with identified putative interacting protein VvPAE, VvPEC, and VvCDP. cYFP empty vector was set as negative control. The arrow indicates the chromogenic location where the target protein and labeled protein co-localize. **D)** Interaction of VvDUF642 and VvPAE was confirmed by Co-IP by transient expression in *N. benthamiana* epidermis.

To understand the role of VvDUF642 in the seed abortion process, we took advantage of mAb techniques by using the VvDUF642 mAb to identify the interacting proteins in grapes. The IP–MS approach led to the identification of 102 putative VvDUF642-interacting proteins (DIPs) (Supplementary Table S2). A gene regulatory network analysis was also performed based on the identified DIPs (Fig. 5A). KEGG enrichment analysis indicated that proteins involved in glycolytic processes, glutamine biosynthesis, plant cell wall biogenesis, carbon metabolism, carbon fixation in photosynthetic organisms, and glyoxylate and dicarboxylate metabolism were significantly enriched (Fig. 5B). Among the DIPs, a group of proteins related to cell wall biogenesis predicted by KEGG enrichment assays, such as pectinesterase, pectin acetylesterase, and alpha-galactosidase, were detected among the putative interacting proteins (Fig. 5A). Split-YFP analyses in *Nicotiana benthamiana* epidermal cells illustrating the putative interaction of VvDUF642 with pectin acetylesterase (VvPAE) and pectinesterase (VvPEC) in the cell wall pattern (Fig. 5C). However, a chloroplast autofluorescence was detected in the VvDUF642 and cellulase domain-containing protein (VvCDP), and VvDUF642 and cYFP empty vector (Fig. 5C). The interaction between VvDUF642 and VvPAE was further validated by Co-IP. The results indicate that VvDUF642 interacts with VvPAE, but not with GFP control (Fig. 5D).

### Overexpression of *VvDUF642* and *VvPAE* inhibits seed production in tomato

**Figure 6.**
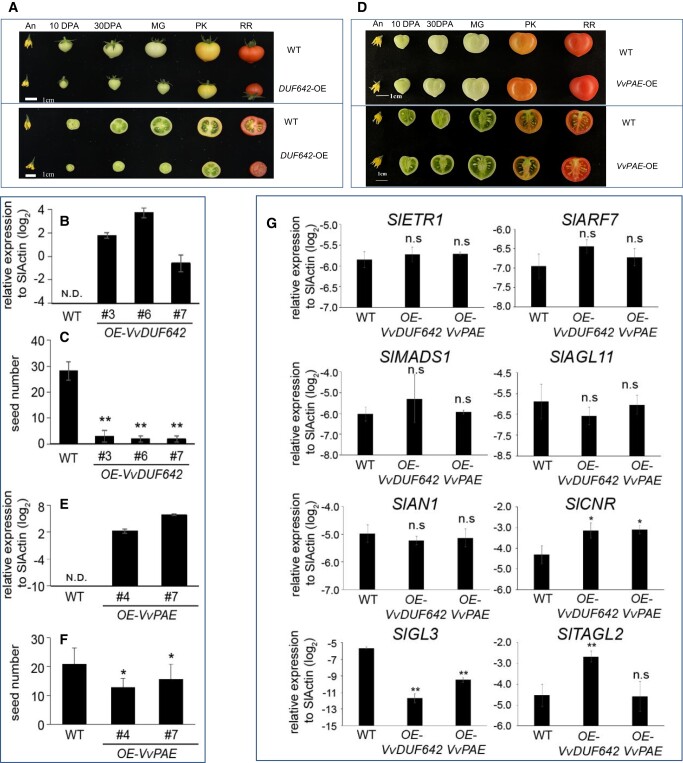
Overexpression of *VvDUF642* and *VvPAE* affects fruit size and seed development in tomato. **A and D)** Fruit phenotype of WT, *VvDUF642*-overexpressing plants (*OE-VvDUF642*, Line #3) and VvPAE-overexpressing plants (*OE-VvPAE*, Line #4) were detected at different developmental stages. An, anthesis; DPA, days post anthesis; MG, mature green; PK, pink; RR, red ripe. **B and E)***VvDUF642* and *VvPAE* expression levels were detected by RT-qPCR. **C and F)** Seed number of WT, *OE-VvDUF642*, and *OE-VvPAE* plants was confirmed. All data are presented as mean ± Sd of biological triplicates (*n* = 3); *P < 0.05; **P < 0.01 (student's *t*-test), **G)** Expression of tomato ripening related genes were confirmed in WT, *OE-VvDUF642* (Line #3), and *OE-VvPAE* (Line #4) plants. n.s., not significant.

Based on the proteomic and WB results, the VvDUF642 protein was highly expressed in the seedless grapes, suggesting that VvDUF642 was likely involved in the seed abortion process. The function of VvDUF642 in seed development was further investigated by ectopic expression in tomato. The *\VvDUF642-*OE plant did not have an altered fruit coloring process (Fig. 6, A and B), but did have significantly lower seed production compared with that in wild-type (WT) tomato (Fig. 6C). These data support our hypothesis that VvDUF642 negatively contributes to seed production, which is consistent with proteomics results.

VvDUF642 directly interacts with VvPAE. To understand whether VvPAE was also involved in seed development, transgenic tomato plants overexpressing *VvPAE* (*VvPAE-*OE) were generated (Fig. 6, D and E). As we have shown, the seed production of *OE-VvPAE* was also significantly reduced compared to that of WT plants (Fig. 6F). These results suggest that VvDUF642 and VvPAE may function together for regulating seed development in plants.

To understand how VvDUF642 and VvPAE affect seed developmental progress, the expression of key regulators involved in tomato fruit development was detected by RT-qPCR. However, most of those genes, including *S. lycopersicum Ethylene receptor 1* (*ETR1*), *S. lycopersicum Auxin response factor 7* (*SlARF7*), *S. lycopersicum MADS-box transcription factor 1* (*SlMADS1*), *S. lycopersicum AGAMOUS-like 11* (*SlAGL11*), and *S. lycopersicum Anthocyanin 1* (*SlAN1*), were not affected (Fig. 6G). However, the expression of *SlCNR* was significantly upregulated when overexpressing *VvDUF642* and *VvPAE* (Fig. 6G). A previous report showed that the absence of *SlCNR* delays fruit ripening and softening as a consequence of reduced ethylene production (Thompson et al. 1999). Moreover, the anthocyanin biosynthesis-related gene *GLABRA3* (*SlGL3*) was downregulated (Fig. 6G), suggesting VvDUF642 and VvPAE may negatively contribute to anthocyanin biosynthesis. Interestingly, the expression of *SlTAGL2* was high in *VvDUF642*-OE plants but not in *VvPAE*-OE plants (Fig. 6G). In Arabidopsis, *TAGL2* was required for the development of floral organs, seeds, and embryos (Flanagan and Ma 1994), suggesting VvDUF642 may also affect seed development through AGL2-related pathway, which may not require VvPAE.

**Figure 7.**
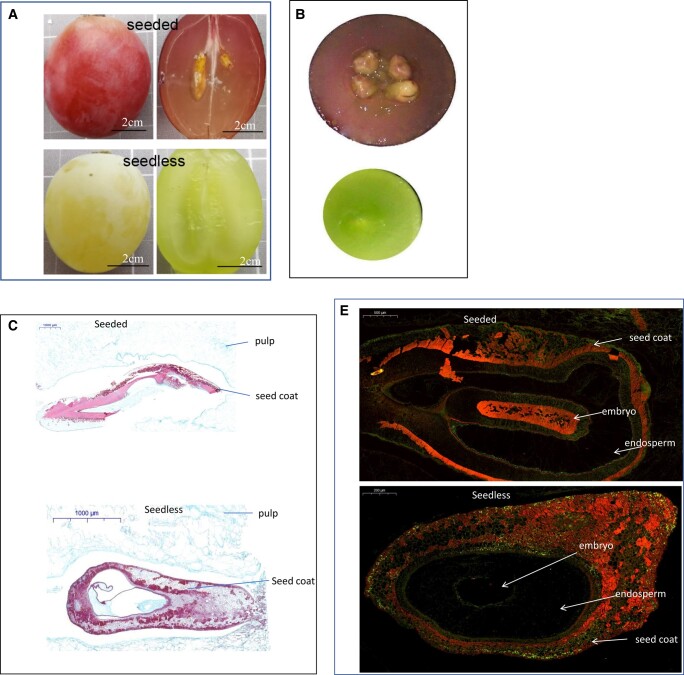

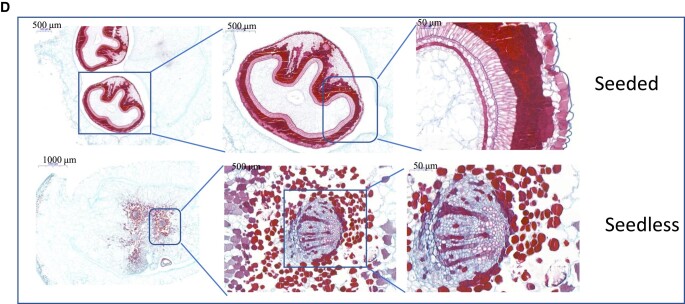
Microscopic analyses of grape seed structure and pectin methyl esterification in seeded and seedless cultivars. **A, B)** Longitudinal (**A**) and transverse (**B**) sections of seeded and seedless grapes at mature stage. **C, D)** Microscopic image of grape seeds from seeded (RG) and seedless (CS) at 35 DPA with HE staining after longitudinal (**C**) and transverse (**D**) sections. **E)** Immunofluorescence assay of VvDUF642 in the seed structure of seeded and seedless grape at 35 DPA with VvDUF642 specific mAb.

Our hypothesis, pectin acetylesterase is related to the degree of acetylation of plant cell wall pectin, thereby impairing the physiochemical properties of the polysaccharides in the cell wall. The cell wall structure of seeded and seedless grapes at the mature stage (Fig. 7A) was analyzed by microscopy with hematoxylin and eosin (HE) staining after longitudinal and transverse sections. A clear cell wall structure of the seed coat, endosperm, and embryo was observed in the seeded grape (Fig. 7, B and C, upper panel). However, in the seedless grape, a thick seed coat, abnormal endosperm, and loss of the embryo were detected (Fig. 7, B and C, lower panel). Immunofluorescence assay with anti-VvDUF642 antibody showed that the VvDUF642 was highly accumulated in the seed coat and endosperm of undeveloped grape seed, but weakly detected in the well-developed seed structures (Fig. 7C). Taken together, our results suggest that the VvDUF642 affects the development of endosperm and seed coat and leads to seedless grapes.

## Discussion

Seed development is a complex process accompanied by significant changes in multiple biological processes. To understand the seed development process in grapes, several large-scale analyses using transcriptome approaches have been performed. Nwafor and colleagues indicated that approximately 80% of annotated grapevine transcripts are commonly expressed in seeded and seedless variants at differential developmental stages, indicating that the major changes may not explain the seed abortion process of grapes (Nwafor et al. 2014). Wang and colleagues investigated the transcriptional differences between the seeded variant RG and the seedless variant CS, illustrating that the genes related to hormone and epigenetic regulation, development of seed coat and endosperm, and formation of seed identity complexes may be involved in grape seed development (Wang et al. 2016). More recently, transcriptome analysis of the F1 generation of RG and CS showed that genes encoding protein kinase, WRKY transcription factors and proteins involved in ethylene/SA/JA signaling, phenylpropanoid biosynthesis, ascorbate and aldarate metabolism, and photosynthesis were upregulated in the seedless plants, whereas genes encoding proteins involved in lignin metabolism, cell growth, phenylpropanoid metabolism, glycolysis/gluconeogenesis, and amino acid biosynthesis were downregulated (Wan et al. 2021). Our proteome analysis exhibited a high correlation with the transcriptomic changes. Proteins related to photosynthesis and oxidative stress were upregulated, and proteins related to glycolysis/gluconeogenesis, amino acid biosynthesis, and photosynthesis were downregulated (Fig. 3, A and B). However, ascorbate and aldarate metabolism-related proteins were downregulated, which was inconsistent with the transcriptional changes (Fig. 3B), suggesting a difference between mRNA and protein levels. Moreover, proteins related to chaperones, oxidative stress, protein folding, endoplasmic reticulum, ATP metabolic process, carbon utilization, and protein stabilization were also significantly changed at the protein level (Fig. 2, D and 3B). Therefore, our proteomic approach provides information regarding the seed development/abortion process in grapes.

A group of proteins was abundantly expressed in the seeded grapes during the full stages of seed development. Most of these proteins were related to the ATP biosynthesis process, including ATP synthesis subunit alpha, ATP synthesis subunit beta, ATP-citrate synthase beta chain protein 2, and ADP/ATP carrier protein 3 (Figs. 2D and 3A). The glycolysis-related proteins included fructose-bisphosphate aldolase 1, pyruvate kinase, and phosphoglucomutase (Figs. 2D and 3B). This is consistent with previous findings showing that ATP and glycolysis are required for seed development in rice (*Oryza sativa*), tomato, and soybean (*Glycine max*) (Smith et al. 1989; She et al. 2010; Wu et al. 2020). These findings suggest that energy production is important for the seed development process.

We also identified proteins that were specifically/highly accumulated in the seedless grapes, including the Grip22-like protein, ras-related protein RABE1c, TPI, DUF642, and 50S ribosomal protein L3, which accumulated only in the seedless grapes (Fig. 2), suggesting the importance of those proteins in the seed abortion process. It was shown that the mRNA level of the Grip22-like protein exhibits dramatic changes during the grape berry ripening process (Davies and Robinson 2000). However, its function in seed development/abortion is still unclear. RABE1c is involved in the degradation of the peroxisomal protein receptor Peroxin7 (PEX7) in Arabidopsis, which is essential for fatty acid β-oxidation and glyoxylate cycle processes (Cui et al. 2013). Overexpression of *GFP-PEX7* leads to defects in peroxisomal β-oxidation in Arabidopsis. These results suggest that peroxisomal function, especially fatty acid β-oxidation, is involved in the seed abortion process, probably through regulation of RABE1c. TPI mediates the reversible inter-conversion of dihydroxyacetone phosphate to glyceraldehyde-3-phosphate, which is a key step in glycolysis, gluconeogenesis and glycerol domain increases carbon metabolism in roots (Dorion et al. 2012; Valancin et al. 2013). These findings illustrate the role of carbon/sugar metabolites in the seed abortion process in grape.

VvDUF642 is one of the proteins highly accumulated in seedless grapes (Fig. 2D). Bioinformatic analysis suggested that there are five genes belonging to the VvDUF642 family in the grapevine genome. Among those, *VvDUF642* was the only gene highly expressed in grape seed, indicating VvDUF642 plays a major role in the regulation of seed development. Although previous reports indicated that DUF642 in different plants participates in the cell wall formation process by interacting with PMEs in the cell wall structure (Vázquez-Lobo et al. 2012; Zúñiga-Sánchez et al. 2014; Palmeros-Suárez et al. 2017; Salazar-Iribe et al. 2017), the biological function of VvDUF642 in grape is still unclear. Pectins are among the major cell wall components of the middle lamella and primary cell wall. In Arabidopsis and tobacco (*Nicotiana tabacum*) pollen tubes, PMEs are predominantly polar localized in the tip region and determine the rigidity of the apical cell wall (Bosch et al. 2005; Jiang et al. 2005; Fayant et al. 2010). PME isoform knockout mutants in Arabidopsis (AtPPME1 or vanguard1) produce unstable pollen tubes that burst when germinated in vitro and have reduced fertilization abilities (Jiang et al. 2005). In Arabidopsis, the *DUF642* family genes positively contribute to silique formation as well as seed development (Zúñiga-Sánchez and Gamboa-de Buen 2012; Zúñiga-Sánchez et al., 2014). However, we provided genetic evidence that VvDUF642 negatively regulates fruit development and seed formation processes (Fig. 4). Although the *DUF642* family genes are highly conserved and exclusively in spermatophytes, they still exhibit their specificities in subcellular localization, tissue specificity, and responses to a different stimulus (Cruz-Valderrama et al. 2019). Therefore, VvDFU642 exhibits a negative role in grape seed development. With the advantage of the mAb library, we further performed IP–MS analysis of VvDUF642-interacting proteins involved in the seed abortion process in grape (Supplementary Table S2). A putative interaction network was further built based on our proteome and IP–MS data. Consistent with the results, pectinesterase and pectin acetylesterase interacted in the network analysis and were enriched in the VvDUF642 co-IP results (Supplementary Fig. S4), suggesting that VvDUF642 may also affect pectin esterase activity in the cell wall structure. This hypothesis was supported by the direct interaction of VvDUF642 and VvPAE in planta (Fig. 5, C and D). Pectin acetylesterase affects the deacetylation of cell wall pectin, therefore modifying the mechanical properties of cell wall polysaccharides (Philippe et al. 2017). Overexpression of *Populus trichocarpa PAE1* decreases the level of acetyl esters of pectin in *N. benthamiana* (Gou et al. 2012). Overexpression of *PtPAE1* in Arabidopsis plants impairs cell elongation, pollen germination, and plant reproduction (Gou et al. 2012). Similarly, overexpression of *VvPAE* affects seed development in tomato (Fig. 6). These findings were supported by the abnormal cell wall structure and a significant increase in pectin methyl esterification in the seedless grapes (Fig. 6), in which VvDUF642 was highly accumulated. Interestingly, several glycoside hydrolase enzymes, such as mannan endo-1,4-beta-mannosidase, alpha-galactosidase, and cellulase domain-containing protein, were also identified. These proteins are also closely related to cell wall structure in different plants (Keegstra and Albersheim 1970). Our findings illustrate the possible role of VvDUF642 in seed abortion probably by interacting with VvPAE, thereby affecting the cell wall development processes. By using the seeded and seedless grape cultivar collections, we found that VvDUF642 was highly accumulated only in the seedless cultivars whereas no signal was observed in the seeded grapes (Fig. 4). These findings provide markers and genetic resources for the breeding of seedless grapes in the future.

The MADS-box family transcription factor is conserved in eukaryotes and functionally important for the regulation of development (Shore and Sharrocks 1995). The *SDI* locus, which harbors the gene encoding MADS-box transcription factor VvAGL11, is the major cause of seedlessness in grape (Royo et al. 2018). In tomato, *SlAGL11*, a homolog of *VvAGL11*, is required for the seed formation process (Ocarez and Mejía 2016). Here we confirmed the expression of *SlAGL11*. However, the expression of *SlAGL11* was not affected by overexpression of *VvDUF642* and *VvPAE* (Fig. 6G), suggesting that the MADS-box gene *VvAGL11* may not be required for VvDUF642- and VvPAE-mediated seed abortion. Interestingly, the expression of *colorless non-ripening* (*CNR*), which is regulated by RIN-MADS, was significantly increased (Fig. 6G). It is known that RIN-MADS is necessary for the tomato ripening process through binding to regulatory genes involved in fruit metabolism, ripening and cell wall modifications (Pesaresi et al. 2014). These results suggest that *SlCNR* may be required for the VvDUF642–VvPAE regulated fruit ripening in tomato. Taken together, in this study, we identified and established a regulation network of seed development-related proteins in grape. The molecular biological and genetic evidence was further provided to validate those regulation networks, which led us to characterize the VvDUF642–VvPAE interacting complex that is required for seed formation. Our data provide potential targets for the breeding of seedlessness grapes and other important fruits in the future.

The mAb-array-based proteomics approach was employed as a high-throughput method for detecting the protein level changes in grapes during seed abortion. This method has been successfully applied in different plants and Mammalia (Wang et al. 2020). Compared with the widely used gel-free quantitative proteomics approach, which identifies and quantifies protein through LC–MS/MS, this mAb-array-based approach has its advantages (Wang et al. 2020; Zeng et al. 2020). For instance, for identifying interacting proteins in planta, a transgenic plant harboring tagged protein needs to be generated which is time-consuming. The specific mAb could be directly used for immunoprecipitation, immunofluorescence labeling, and fluorescence-activated cell sorting through flow cytometry without generating transgenic plants which is time-consuming. However, the mAb-array-based method is relatively high costing for antibody generation, antibody array construction, and protein identification by MS. However, once the mAb library was generated, it could be used for the rapid screening of proteins for different purposes, such as plant development, and responses to biotic and abiotic stresses.

## Materials and methods

### Plant materials and growth conditions

Grapevines (*V. vinifera*) were grown in the Zhengzhou National Germplasm Nursery (113.65 34:76) in China. Samples were collected from 1 May to 5 June, 3, 15, 25, and 35 DPA. Each seed (aborting seed) was collected from a grape berry. The collected samples were frozen in liquid nitrogen and stored at −80 °C until protein extraction. All experiments were performed in three biological replicates, and each replicate included at least 10 berries.

### Protein extraction

Samples of grape tissues were ground into a fine powder with liquid nitrogen in the presence of 2% v/v polyvinylpolypyrrolidone. Five volumes of ice-cold extraction buffer (200 mM Tris–HCl (pH 8.0), 200 mM NaCl, 50 mM sodium ascorbate, 5 mM EDTA, 10% v/v glycerol, 1% v/v NP-40, 1% v/v Triton X-100, 1% v/v Tween-20, 0.25% w/v sodium metabisulfite, protease inhibitor cocktail, and 2 mM PMSF) were added to each sample, and the samples were rotated at 4 °C for 2 h. The mixtures were centrifuged at 15,000 × *g* for 30 min, and supernatants were collected by filtration.

### mAb library construction

mAbs were produced using a standard method. First, the proteins extracted from the mesocarp of grape berries and leaves were mixed with an equal volume of complete Freund's adjuvant to prepare the antigens. Three BALB/c mice were immunized four times, with 14-d intervals, using an initial 60 *μ*g of antigens followed by booster injections of 30 *μ*g of antigens. Polyethylene glycol was used as the adjuvant during the second and subsequent immunizations. Mouse spleen cells (2.0 × 10^7^ mL^−1^) were isolated and fused with mouse P3X63Ag8.653 cells to generate hybridoma cells, which were subcloned by limiting dilution. An enzyme-linked immunosorbent assay was used to verify that the hybridoma cell clones produced IgG. Positive clones were then used in expansion cultures. Antibodies were purified from culture medium supernatants using protein G.

### Antibody microarray screening

Each antibody was added (10 nL spot^−1^) to the surface of nitrocellulose-coated microscope slides (FAST chip, CapitalBio, Beijing, China) using an Inkjet Microarrayer (Arrayjet Ltd., Roslin, UK) at 4 °C. The prepared slides were stored at −80 °C until use. For array screening, grape proteins (0.1 *μ*g μL^−1^) were labeled with NHS-biotin (Molecular Probes, Carlsbad, CA). The microarray slides were washed twice with TBS washing solution (TBS supplemented with 0.1% v/v Tween 20) and then blocked with 10% w/v BSA in TBS washing solution at 4 °C overnight. The slides were washed four times for 15 min each with TBS washing solution and then incubated with 30 *μ*g of biotin-labeled samples (30 mL final volume) at room temperature for 1 h. The slides were washed four times with TBS washing solution and then incubated with Cy3-labeled streptavidin (1:5,000) at room temperature for 1 h. After four washes with TBS washing solution and four washes with double-distilled H_2_O, the slides were centrifuged at 1,500 × *g* for 2 min to remove any residual buffer and dried. The dried slides were scanned with a GenePix microarray scanner (Axon Instruments, Union City, CA, USA). The Cy3 fluorescence signals were obtained by excitation with a 532 nm laser and detected at 570 nm.

### Processing of MabArray data

The Cy3 fluorescence intensity was quantified using the GenePix 6.0 program (Axon Instruments) and further analyzed with the limma package (http://bioconductor.org/). Original data were normalized by background correction and quantile normalization methods using the “background Correct” and “normalize Between Arrays” functions, respectively. Hierarchical clustering and principal component analysis were used to assess the reproducibility of the microarray data in the three biological replicates. A linear model was applied to analyze the differentially expressed proteins in the antibody microarray with the “lmFit” and “eBayes” functions. A fold-change cutoff of 1.5 (0.585 for log_2_ transformed data) and a *P*-value cutoff of 0.05 were applied.

### Sample preparation for mass spectrometry

Collected cells were washed three times with phosphate-buffered saline (PBS) to remove serum proteins and then lysed in buffer containing 7 M urea, 2% w/v SDS, 0.1% w/v PMSF, and 65 mM DTT. The sample was sonicated in ice water and centrifuged at 14,000 × *g* for 30 min at 4 °C to collect the supernatant. The protein concentration was determined with the BCA assay. Fifty micrograms of protein extracted from cells were suspended in 50 *μ*L of the solution, reduced by adding 1 *μ*L of 1 M dithiothreitol at 55 °C for 1 h and alkylated by adding 5 *μ*L of 20 mM iodoacetamide in the dark at 37 °C for 1 h. Then, the sample was precipitated using 300 *μ*L of prechilled acetone at −20 °C overnight. The precipitate was washed twice with cold acetone and then resuspended in 50 mM ammonium bicarbonate. Finally, the proteins were digested with sequence-grade modified trypsin (Promega, Madison, WI) at a substrate/enzyme ratio of 50:1 (w/w) at 37 °C for 16 h.

### LC–MS/MS analysis

The trypsin-digested were analyzed by nLC–MS/MS (Dionex Ultimate 3000 RLSCnano System) interfaced with Eclipse Tribrid mass spectrometer (Thermofisher). Samples were loaded onto a fused silica trap column Acclaim PepMap 100, 75 *μ*m × 2 cm (ThermoFisher). After washing for 5 min at 5 *μ*L min^−1^ with 0.1% v/v TFA, the trap column was brought in line with an analytical column (Nanoease MZ peptide BEH C18, 130 A, 1.7 *μ*m, 75 *μ*m × 250 mm, Waters Corporation, MA, USA) for LC–MS/MS. Peptides were fractionated at 300 nL min^−1^ using a segmented linear gradient 4% to 15% solution B in 30 min (where solution A: 0.2% formic acid, and solution B: 0.16% formic acid, 80% acetonitrile), 15% to 25% solution B in 40 min, 25% to 50% solution B in 44 min, and 50% to 90% solution B in 11 min. Solution B then returns at 4% for 5 min for the next run. The scan sequence began with an MS1 spectrum (Orbitrap analysis, resolution 120,000, scan range from *m*/*z* 350 to 1,600, automatic gain control (AGC) target 1E6, maximum injection time 100 ms). The top S (3 s) and dynamic exclusion of 60 s were used for the selection of Parent ions for MS/MS. Parent masses were isolated in the quadrupole with an isolation window of 1.4 *m*/*z*, AGC target 1E5, and fragmented with higher-energy collisional dissociation with a normalized collision energy of 30%. The fragments were scanned in Orbitrap with a resolution of 30,000. The MS/MS scan range was determined by the charge state of the parent ion but a lower limit was set at 100 amu.

UniProtKB *V. vinifera* database [containing 29,760 entries] was set up for data searching, and trypsin was set as the digestion enzyme. Peptides showing a false discovery rate <0.01 were removed from the analysis. PEAKS Q was used to calculate peptide and protein abundances in the differential IP experiment. Data were normalized using the median peptide abundances. Proteins with an abundance fold-change >2.0 (*P* < 0.05; analysis of variance) were designated as differentially expressed proteins.

### High-pH reversed-phase separation

The peptide mixture of two cell lines (K&M) was redissolved in buffer A (20 mM ammonium formate in H_2_O (pH 10.0), adjusted with ammonium hydroxide) and then fractionated by high-pH separation using an Ultimate 3000 system (Thermo Fisher Scientific, MA, USA) connected to a reversed-phase column (XBridge C18 column, 4.6 mm × 250 mm, 5 *μ*m, Waters Corporation, MA, USA). High-pH separation was performed using a linear gradient, starting from 5% to 45% buffer B (20 mM ammonium formate in 80% (v/v) acetonitrile (pH 10.0), adjusted with ammonium hydroxide) in 40 min. The column was re-equilibrated at the initial condition for 15 min with a flow rate of 1 mL min^−1^ at 30 °C. Ten fractions were collected; each fraction was dried in a vacuum concentrator for the next step.

### Western blotting analysis

Proteins (20 *μ*g) from each sample were separated by SDS-PAGE and then transferred to polyvinylidene fluoride membranes. The membranes were incubated with appropriate primary antibodies and an HRP-conjugated antimouse IgG secondary antibody. Immune-reactive bands were visualized with an enhanced chemiluminescence system and exposed to X-ray film. Signal intensities were quantified by the ImageJ program and normalized against the corresponding β-actin signal.

### GO enrichment analysis

GO enrichment was performed by using the KEGG tool. Briefly, all genes were annotated against the v1 version of the 12× draft annotation of the grapevine genome using the CRIBI Biotechnology Center website (http://genomes.cribi.unipd.it/) combined with the grapevine molecular network VitisNet (Grimplet et al. 2012). Next, all differentially expressed genes for both genotypes were input into the AgriGO analysis tool (Du et al. 2010). Using a hypergeometric test, a GO term was considered significantly enriched if the false discovery rate (FDR) was <0.05 and the *P*-value was <0.01 when compared to all gene transcripts annotated in the reference genome (supported in AgriGO). Furthermore, the REVIGO web server (http://revigo.irb.hr/) was used to summarize the processes represented in the lists of significantly enriched GO terms by removing redundant terms as described previously (Sweetman et al. 2012).

### IP–MS assays

Total dairy cow protein samples (1 mg) were precleared with protein G resin (Invitrogen, Carlsbad, CA, USA) at 4 °C for 4 h and then incubated with a CNBr-conjugated peach antibody overnight. After extensive washes, the IP complexes were eluted with 0.2 M glycine (pH 2.5) and analyzed by SDS-PAGE followed by silver staining or Western blotting. Protein bands from the silver-stained gels corresponding to the visualized band in the WB were excised for in-gel trypsin digestion and MS analysis. For the co-IP assay, proteins were incubated with the same antibodies used for the IP assays. After extensive washes, the IP complexes were eluted with 0.2 M glycine (pH 2.5) and subjected to in-solution trypsin digestion and MS analysis. Anti-mouse IgG was used as a control.

### Establishment of PPI networks

Interacting proteins were enriched by immunoprecipitation with individual specific antibodies as described. The putative interacting protein was identified by MS/MS after in-solution digestion with trypsin. PPI networks from grapevine were obtained by aligning the protein sequences of grapevine with the Arabidopsis database in the STRING using diamond (version: v2.0.7), and the R package org.At.tair.db (version 3.16.0) and clusterProfiler (version 4.6.0) were used for GO and KEGG enrichment analysis.

### Plasmid construction and plant transformation

To generate *VvDUF642*-overexpressing tomato (*S. lycopersicum*) plants, full-length *VvDUF642* cDNA was amplified and cloned into the pBWA(V)HS vector (Supplementary Fig. S4). The construct was then transformed into Agrobacterium and introduced into tomato plants by Agrobacterium-mediated transformation (Van Eck et al. 2019).

### *Solanum lycopersicum* “MicroTom” growth environment

Plants were grown in a controlled environment growth chamber with the following settings: Temperature: 24 ± 2 °C (day) and 18C ± 2 °C (night). Relative humidity: 60 ± 5%. Photoperiod: 16 h of light and 8 h of darkness.

### Seed sectioning and microscopic analysis

The seeds (aborting seeds) were fixed, embedded in wax, and sectioned as described previously for plant materials (Godel-Jędrychowska et al. 2019). The seed sections were washed gently in distilled water, and the nuclei were stained in a hematoxylin staining solution (Sangon Biotech). The stained seed sections were rinsed with tap water, differentiated with 0.1% v/v hydrochloric acid–ethanol, and rinsed with tap water. For eosin staining, the seed sections were washed in PBS/PBST, rinsed in tap water and 95% alcohol, and then counterstained with an eosin staining solution (Sangon Biotech). The stained sections were dehydrated through 95% alcohol and cleared in xylene. Finally, the stained plates were mounted and detected under microscopy.

For pectin detection, seed sections were incubated first with 3% (w/v) MP/PBS and incubated with a 100-fold dilution of LM19 monoclonal antibody (PlantProbes) in 3% MP/PBS. Samples were then washed three times with PBS and incubated with 100-fold diluted anti-rat IgG linked to FITC in MP/PBS in the dark. Finally, the slides were washed in blocking buffer, rinsed in PBS and sterile distilled water, and mounted with a fluoromount aqueous mounting medium (Sigma). The sections were examined with an epifluorescence microscope (Nikon Eclipse Ti-SR) equipped with a filter set for visualization of Alexa Fluor 488 (excitation filter BP465–495, dichromatic mirror DM500, barrier filter BA520IF). Images were captured with a Nikon DS-U3 camera.

### Bimolecular fluorescence complementation assays

The full-length coding sequences of *DUF642*, *Pectin acetylesterase* (*PAC*, VIT-02s0087g00400), *Pectinesterase* (*PEC*, VIT-07s0005g00730), and *Cellulase domain-containing protein* (*CDP*, VIT-18s0001g12830) were cloned into pEarleyGate201-NYFP and pEarleyGate202-CYFP, which were carrying nYFP-protein and cYFP-protein, respectively. The constructs were then transformed into *Agrobacterium tumefaciens* GV3101. Leaves of 5-wk-old *N. benthamiana* were co-infiltrated with strains carrying different constructs.

Fluorescence microscopy was conducted using a fluorescence microscope or confocal laser scanning microscope (LEICA TCS SP8 X, Leica, Wetzlar, Germany). The maximum excitation wavelength/maximum emission wavelength of EYFP and TagBFP2 were 513/527 and 399 nm/454 nm, respectively. If the microscope did not have a special optical filter for EYFP, the FITC channel could be used as a substitute. The DAPI channel was suitable for observing TagBFP2 and DAPI.

### Protein extraction and co-IP assay

Total protein was extracted from *N. benthamiana* leaves transiently expressing *DUF642*-flag, *VvPAE*-HA, and *GFP*-flag using extraction buffer (100 mM Tris-Cl pH7.5, 150 mM NaCl, 0.5% v/v NP-40, 1 mM EDTA, 1 mM DTT, and protease inhibitor cocktail). Extracted proteins were incubated with an antiflag antibody fused to protein A agarose bead (Invitrogen) at 4 °C for 2 h. After washing with 1× PBS, the protein was separated on 12% SDS-PAGE and followed by immunoblotting with antiflag and anti-HA antibodies.

### Gene expression analysis by RT-qPCR

For RT-qPCR, the cDNAs were synthesized from1 *µ*g of total RNA using FastKing RT Kit with DNase (Tiangen Biotech, Beijing, PRC). The gene-specific primers were designed using DNAMAN software. PCR reactions with the Roche FastStart DNA Master SYBR Green I reagent were performed on Roche LightCycler480 instruments with the following procedure: 95 °C for 5 min, followed by 45 cycles of 95 °C for 10 s, 60 °C for 10 s, and 72 °C for 20 s. The tomato *SIActin* gene was used as the internal control. The relative gene expression level was analyzed by the 2-ΔΔCT method. The used primers are listed in Supplementary Table S3.

### Accession numbers

Sequence data from this article can be found in the GenBank/EMBL data libraries under the following accession numbers: VvDUF642: Protein ID, F6H3T7; Gene ID, 100252479; VvPAE: Protein ID, D7TFE6; VvPEC: Protein ID, F6HZ64; VvCDP: Protein ID, E0CQG0; *SlARF7*:Gene ID, 100191131; *SlGL3*: Gene ID, 101254527; *SICNR*: Gene ID, 101256245; *SIETR1*: Gene ID, 606298; *SIAGL*: Gene ID, 543841; *SIAN1*: Gene ID, 101262902; *SITAGL2*: Gene ID, 543770; *SIMADS1*: Gene ID, 543840.

## Supplementary Material

kiad682_Supplementary_Data

## Data Availability

The data underlying this article will be shared on reasonable request to the corresponding author.

## Supplementary data

The following materials are available in the online version of this article.

**Supplementary Figure S1.** Protein quality validation.

**Supplementary Figure S2.** Antibody quality was validated by WB analyses with individual antibodies.

**Supplementary Figure S3.** PPI network of differentially expressed proteins by IP–MS.

**Supplementary Figure S4.** Tomato expression vector constructed.

**Supplementary Table S1.** Proteins identified by IP and MS.

**Supplementary Table S2.** List of proteins identified by VvDUF642 IP–MS.

**Supplementary Table S3.** Primers used in this study.

## References

[kiad682-B1] Borner GHH , Lilley KS, Stevens TJ, Dupree P. Identification of glycosylphosphatidylinositol-anchored proteins in Arabidopsis. A proteomic and genomic analysis. Plant Physiol. 2003:132(2):568–577. 10.1104/pp.103.021170 12805588 PMC166998

[kiad682-B2] Bosch M , Cheung AY, Hepler PK. Pectin methylesterase, a regulator of pollen tube growth. Plant Physiol. 2005:138(3):1334–1346. 10.1104/pp.105.059865 15951488 PMC1176407

[kiad682-B3] Cai Y , Yin L, Tu W, Deng Z, Yan J, Dong W, Gao H, Xu J, Zhang N, Wang J, et al Ectopic expression of *VvSUC27* induces stenospermocarpy and sugar accumulation in tomato fruits. Front Plant Sci. 2021:12:759047. 10.3389/fpls.2021.759047 34868153 PMC8637806

[kiad682-B4] Calderan-Rodrigues MJ , Guimarães Fonseca J, de Moraes FE, Vaz Setem L, Carmanhanis Begossi A, Labate CA. Plant cell wall proteomics: a focus on monocot species, *Brachypodium distachyon. Saccharum spp.*and*Oryza sativa*. Int J Mol Sci. 2019:20(8):1975. 10.3390/ijms20081975 31018495 PMC6514655

[kiad682-B5] Cruz-Valderrama JE , Gómez-Maqueo X, Salazar-Iribe A, Zúñiga-Sánchez E, Hernández-Barrera A, Quezada-Rodríguez E, Gamboa-deBuen A. Overview of the role of cell wall DUF642 proteins in plant development. Int J Mol Sci. 2019:20(13):3333. 10.3390/ijms20133333 31284602 PMC6651502

[kiad682-B6] Cui S , Fukao Y, Mano S, Yamada K, Makoto H, Mikio N. Proteomic analysis reveals that the Rab GTPase RabE1c is involved in the degradation of the peroxisomal protein receptor PEX7 (peroxin 7). J Biol Chem. 2013:288(8):6014–6023. 10.1074/jbc.M112.438143 23297417 PMC3581416

[kiad682-B7] Davies C , Robinson SP. Differential screening indicates a dramatic change in mRNA profiles during grape berry ripening. Cloning and characterization of cDNAs encoding putative cell wall and stress response proteins. Plant Physiol. 2000:122(3):803–812. 10.1104/pp.122.3.803 10712544 PMC58916

[kiad682-B8] Dorion S , Clendenning A, Jeukens J, Salas JJ, Parveen N, Haner AA, Law RD, Force EM, Rivoal J. A large decrease of cytosolic triosephosphate isomerase in transgenic potato roots affects the distribution of carbon in primary metabolism. Planta. 2012:236(4):1177–1190. 10.1007/s00425-012-1675-1 22678033

[kiad682-B9] Du Z , Zhou X, Ling Y, Zhang Z, Su Z. AgriGO: a GO analysis toolkit for the agricultural community. Nucleic Acids Res. 2010:38(suppl_2):W64–W70. 10.1093/nar/gkq310 20435677 PMC2896167

[kiad682-B10] Fayant P , Girlanda O, Chebli Y, Aubin C-E, Villemure I, Geitmann A. Finite element model of polar growth in pollen tubes. Plant Cell. 2010:22(8):2579–2593. 10.1105/tpc.110.075754 20699395 PMC2947179

[kiad682-B11] Flanagan CA , Ma H. Spatially and temporally regulated expression of the MADS-box gene AGL2 in wild-type and mutant arabidopsis flowers. Plant Mol Biol. 1994:26(2):581–595. 10.1007/BF00013745 7948914

[kiad682-B12] Fujita SC , Zipursky SL, Benzer S, Ferrús A, Shotwell SL. Monoclonal antibodies against the *Drosophila nervous* system. Proc Natl Acad Sci USA. 1982:79(24):7929–7933. 10.1073/pnas.79.24.7929 6818557 PMC347463

[kiad682-B13] Gao J , Gao Y, Ju Y, Yang J, Wu Q, Zhang J, Du X, Wang Z, Song Y, Li H, et al Proteomics-based generation and characterization of monoclonal antibodies against human liver mitochondrial proteins. Proteomics. 2006:6(2):427–437. 10.1002/pmic.200500409 16342244

[kiad682-B14] Godel-Jędrychowska K , Maćkowska K, Kurczyńska E, Grzebelus E. Composition of the reconstituted cell wall in protoplast-derived cells of daucus is affected by phytosulfokine (PSK). Int J Mol Sci. 2019:20(21):5490. 10.3390/ijms20215490 31690047 PMC6862203

[kiad682-B15] Gou JY , Miller LM, Hou G, Yu XH, Chen XY, Liu CJ. Acetylesterase-mediated deacetylation of pectin impairs cell elongation, pollen germination, and plant reproduction. Plant Cell. 2012:24(1):50–65. 10.1105/tpc.111.092411 22247250 PMC3289554

[kiad682-B16] Grimplet J , Van Hemert J, Carbonell-Bejerano P, Díaz-Riquelme J, Dickerson J, Fennell A, Pezzotti M, Martínez-Zapater JM. Comparative analysis of grapevine whole-genome gene predictions, functional annotation, categorization and integration of the predicted gene sequences. BMC Res Notes. 2012:5(1):213. 10.1186/1756-0500-5-213 22554261 PMC3419625

[kiad682-B17] Guergova-Kuras M , Kurucz I, Hempel W, Tardieu N, Kadas J, Malderez-Bloes C, Jullien A, Kieffer Y, Hincapie M, Guttman A, et al Discovery of lung cancer biomarkers by profiling the plasma proteome with monoclonal antibody libraries. Mol Cell Proteomics. 2011:10(12):M111.010298. 10.1074/mcp.M111.010298 PMC3237079 21947365

[kiad682-B18] Jamet E , Canut H, Boudart G, Pont-Lezica RF. Cell wall proteins: a new insight through proteomics. Trends Plant Sci. 2006:11(1):33–39. 10.1016/j.tplants.2005.11.006 16356755

[kiad682-B19] Jiang L , Yang S-L, Xie L-F, Puah CS, Zhang X-Q, Yang W-C, Sundaresan V, Ye D. VANGUARD1 encodes a pectin methylesterase that enhances pollen tube growth in the Arabidopsis style and transmitting tract. Plant Cell. 2005:17(2):584–596. 10.1105/tpc.104.027631 15659637 PMC548828

[kiad682-B20] Joldersma D , Liu Z. The making of virgin fruit: the molecular and genetic basis of parthenocarpy. J Exp Bot. 2018:69(5):955–962. 10.1093/jxb/erx446 29325151 PMC6018997

[kiad682-B21] Keegstra K , Albersheim P. The involvement of glycosidases in the cell wall metabolism of suspension-cultured Acer pesudoplatanus cells. Plant Physiol. 1970:45(6):675–678. 10.1104/pp.45.6.675 16657372 PMC396491

[kiad682-B22] Keller M . The chapter 6—developmental physiology. In: Keller M, editors. The science of grapevines. Academic Press; 2015. p. 169–225.

[kiad682-B23] Korkutal I . Embryo abortion in some new seedless table grape (*Vitis vinifera* L.) varieties. Int J Bot. 2005:1(1):1–4. 10.3923/ijb.2007.128.128

[kiad682-B24] Lahogue F , This P, Bouquet A. Identification of a codominant scar marker linked to the seedlessness character in grapevine. Theor Appl Genet. 1998:97(5–6):950–959. 10.1007/s001220050976

[kiad682-B25] Li Z , Jiao Y, Zhang C, Dou M, Weng K, Wang Y, Xu Y. *VvHDZ28* positively regulate salicylic acid biosynthesis during seed abortion in thompson seedless. Plant Biotechnol J. 2021:19(9):1824–1838. 10.1111/pbi.13596 33835678 PMC8428834

[kiad682-B26] Nwafor CC , Gribaudo I, Schneider A, Wehrens R, Grando MS, Laura Costantini L. Transcriptome analysis during berry development provides insights into co-regulated and altered gene expression between a seeded wine grape variety and its seedless somatic variant. BMC Genomics. 2014:15(1):1030. 10.1186/1471-2164-15-1030 25431125 PMC4301461

[kiad682-B27] Ocarez N , Mejía N. Suppression of the D-class MADS-box AGL11 gene triggers seedlessness in fleshy fruits. Plant Cell Rep. 2016:35(1):239–254. 10.1007/s00299-015-1882-x 26563346

[kiad682-B28] Palmeros-Suárez PA , Massange-Sánchez JA, Sánchez-Segura L, Martínez-Gallardo NA, Espitia Rangel E, Gómez-Leyva JF, Délano-Frier JP. AhDGR2, an amaranth abiotic stress-induced DUF642 protein gene, modifies cell wall structure and composition and causes salt and ABA hyper-sensibility in transgenic Arabidopsis. Planta. 2017:245(3):623–640. 10.1007/s00425-016-2635-y 27988887

[kiad682-B29] Pesaresi P , Mizzotti C, Colombo M, Masiero S. Genetic regulation and structural changes during tomato fruit development and ripening. Front Plant Sci. 2014:5:124. 10.3389/fpls.2014.00124 24795731 PMC4006027

[kiad682-B30] Philippe F , Pelloux J, Rayon C. Plant pectin acetylesterase structure and function: new insights from bioinformatic analysis. BMC Genomics. 2017:18(1):456. 10.1186/s12864-017-3833-0 28595570 PMC5465549

[kiad682-B31] Royo C , Torres-Pérez R, Mauri N, Diestro N, Cabezas JA, Marchal C, Lacombe T, Ibáñez J, Tornel M, Carreño J, et al The major origin of seedless grapes is associated with a missense mutation in the MADS-box gene *Vvi ALG11*. Plant Physiol. 2018:177(3):1234–1253. 10.1104/pp.18.00259 29853599 PMC6053000

[kiad682-B32] Salazar-Iribe A , Zúñiga-Sánchez E, Mejía EZ, Gamboa-deBuen A. Cell wall localization of two DUF642 proteins. BIIDXI and TEEBE, during *Meloidogyne incognita* early inoculation. Plant Pathol J. 2017:33(6):614–618. 10.5423/PPJ.NT.05.2017.0101 29238286 PMC5720610

[kiad682-B33] She KC , Kusano H, Yaeshima M, Sasaki T, Satoh H, Shimada H. Reduced rice grain production under high-temperature stress closely correlates with ATP shortage during seed development. Plant Biotechnol. 2010:27(1):67–73. 10.5511/plantbiotechnology.27.67

[kiad682-B34] Shi Q , Zhou L, Wang Y, Ma H. A strategy for screening monoclonal antibodies for Arabidopsis flowers. Front Plant Sci. 2017:8:270. 10.3389/fpls.2017.00270 28293248 PMC5330178

[kiad682-B35] Shore P , Sharrocks AD. The MADS-box family of transcription factors. Eur J Biochem. 1995:229(1):1–13. 10.1111/j.1432-1033.1995.tb20430.x 7744019

[kiad682-B36] Smith AJ , Rinne RW, Seif RD. Phosphoenolpyruvate carbixylase and pyruvate kinase involvement in protein and oil biosynthesis during soybean seed development. Crop Sci. 1989:29(2):349–353. 10.2135/cropsci1989.0011183X002900020024x

[kiad682-B901] Sreenivasulu N , Wobus U. Seed-development programs: a systems biology-based comparison between dicots and monocots. Annu Rev Plant Biol. 2013:64:189–217. 23451786 10.1146/annurev-arplant-050312-120215

[kiad682-B37] Sweetman C , Wong DC, Ford CM, Drew DP. Transcriptome analysis at four developmental stages of grape berry (*Vitis vinifera* cv. *Shiraz*) provides insights into regulated and coordinated gene expression. BMC Genomics. 2012:13(1):691. 10.1186/1471-2164-13-691 23227855 PMC3545830

[kiad682-B38] Thompson A , Tor M, Barry C, Vrebalov J, Orfifila C, Jarvis M, Giovannoni JJ, Grierson D, Seymour GB. Molecular and genetic characterization of a novel pleiotropic tomato-ripening mutant. Plant Physiol. 1999:120(2):383–390. 10.1104/pp.120.2.383 10364389 PMC59276

[kiad682-B39] Uhlen M , Fagerberg L, Hallstrom BM, Lindskog C, Oksvold P, Mardinoglu A, Sivertsson Å, Kampf C, Sjöstedt E, Asplund A, et al Proteomics. Tissue-based map of the human proteome. Science. 2015:347(6220):1260419. 10.1126/science.1260419 25613900

[kiad682-B40] Valancin A , Srinivasan B, Rivoal J, Jolicoeur M. Analyzing the effect of decreasing cytosolic triosephosphate isomerase on *Solanum tuberosum* hairy root cells using a kinetic-metabolic model. Biotechnol Bioeng. 2013:110(3):924–935. 10.1002/bit.24747 23055265

[kiad682-B41] Van Eck J , Keen P, Tjahjadi M. *Agrobacterium tumefacienc*-mediated transformation of tomato. Methods Mol Biol. 2019:1864:225–234. 10.1007/978-1-4939-8778-8_16 30415340

[kiad682-B42] Vázquez-Lobo A , Roujol D, Zúñiga-Sánchez E, Albenne C, Pinero D, Gamboa-deBuen A, Jamet E. The highly conserved spermatophyte cell wall DUF642 protein family: phylogeny and first evidence of interaction with cell wall polysaccharides *in vitro*. Mol Phylogenet Evol. 2012:63(2):510–520. 10.1016/j.ympev.2012.02.001 22361214

[kiad682-B43] Wan R , Guo C, Hou X, Zhu Y, Gao M, Hu X, Zhang S, Jiao C, Guo R, Li Z, et al Comparative transcriptomic analysis highlights contrasting levels of resistance of *Vitis vinifera* and *Vitis amurensis* to *Botrytis cinerea*. Hortic Res. 2021:8(1):103. 10.1038/s41438-021-00537-8 33931625 PMC8087793

[kiad682-B44] Wang D , Hincapie M, Guergova-Kuras M, Kadas J, Takacs L, Karger BL. Antigen identification and characterization of lung cancer specific monoclonal antibodies produced by mAb proteomics. J Proteome Res. 2010:9(4):1834–1842. 10.1021/pr900997z 20146545 PMC2849899

[kiad682-B45] Wang L , Hu X, Jiao C, Li Z, Fei Z, Yan X, Liu C, Wang Y, Wang X. Transcriptome analyses of seed development in grape hybrids reveals a possible mechanism influencing seed size. BMC Genomics. 2016:17(1):898. 10.1186/s12864-016-3193-1 27829355 PMC5103508

[kiad682-B46] Wang Z , Li Y, Hou B, Pronobis MI, Wang M, Wang Y, Chen G, Wen W, Wang Y, Tang Y, et al An array of 60,000 antibodies for proteome-scale antibody generation and target discovery. Sci Adv. 2020:6:eaax2271. 10.1126/sciadv.aax2271 32195335 PMC7065887

[kiad682-B47] Wu YJ , Chen HM, Wu TT, Wu JS, Chu RM, Juang RH. Preparation of monoclonal antibody bank against whole water-soluble proteins from rapid-growing bamboo shoots. Proteomics. 2006:6(22):5898–5902. 10.1002/pmic.200600278 17051642

[kiad682-B48] Wu TY , Müller M, Gruissem W, Bhulla NK. Genome wide analysis of the transcriptional profiles in different regions of the developing rice grains. Rice (N Y). 2020:13(1):62. 10.1186/s12284-020-00421-4 32894395 PMC7477059

[kiad682-B49] Xie X , Wang Y. *VqDUF642*, a gene isolated from the Chinese grape *Vitis quinquangularis*, is involved in berry development and pathogen resistance. Planta. 2016:244(5):1075–1094. 10.1007/s00425-016-2569-4 27424038

[kiad682-B50] Zeng W , Niu L, Wang Z, Wang X, Wang Y, Pan L, Lu Z, Cui G, Weng W, Wang M, et al Application of an antibody chip for screening differentially expressed proteins during peach ripening and identification of a metabolon in the SAM cycle to generate a peach ethylene biosynthesis model. Hortic Res. 2020:7(1):31. 10.1038/s41438-020-0249-9 32194967 PMC7072073

[kiad682-B51] Zúñiga-Sánchez E , Gamboa-de Buen A. The two DUF642 At5g11420 and At4g32460-encoded proteins interact in vitro with the AtPME3 catalytic domain. In: Cai J Wang R, editors. Protein interactions. London, UK: IntechOpen; 2012. p. 119–142.

[kiad682-B52] Zúñiga-Sánchez E , Soriano D, Martínez-Barajas E, Orozco-Segovia1 A, Gamboa-deBuen A. BIIDXI, the Ag4g32460 DUF642 gene, is involved in pectin methyl esterase regulation during *Arabidopsis thaliana* seed germination and plant development. BMC Plant Biol. 2014:14(1):338. 10.1186/s12870-014-0338-8 25442819 PMC4264326

