# Supplementary material for: Antibody array-based proteome approach reveals proteins involved in grape seed development
Source: Plant Physiol. 2024 Feb 23;195(1):462–78. doi: 10.1093/plphys/kiad682 (PMC11060674; doi:10.1093/plphys/kiad682)
Supplement: kiad682_Supplementary_Data [file kiad682_supplementary_data.zip › 1Supplemental Figure S14.pdf]

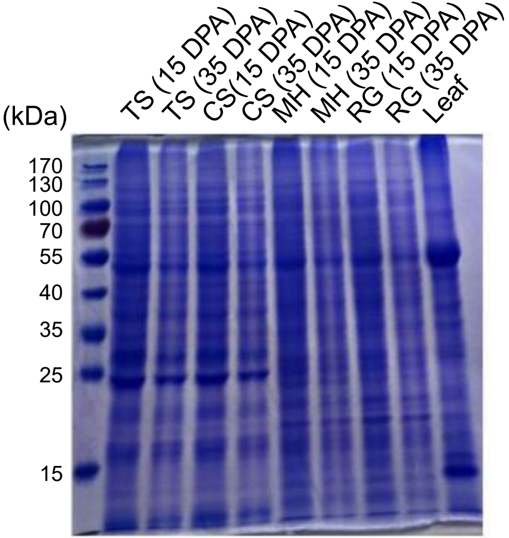

Supplemental Figure S1. Protein quality validation.

The total protein extracted from grape berries and leaves was separated using a 12% SDS-PAGE and stained with Coomassie Brilliant Blue. Samples included Thompson Seedless (TS), Centennial Seedless (CS), Muscat Hamburg (MH), Red Global (RG), and were collected at different days post anthesis (DPA).

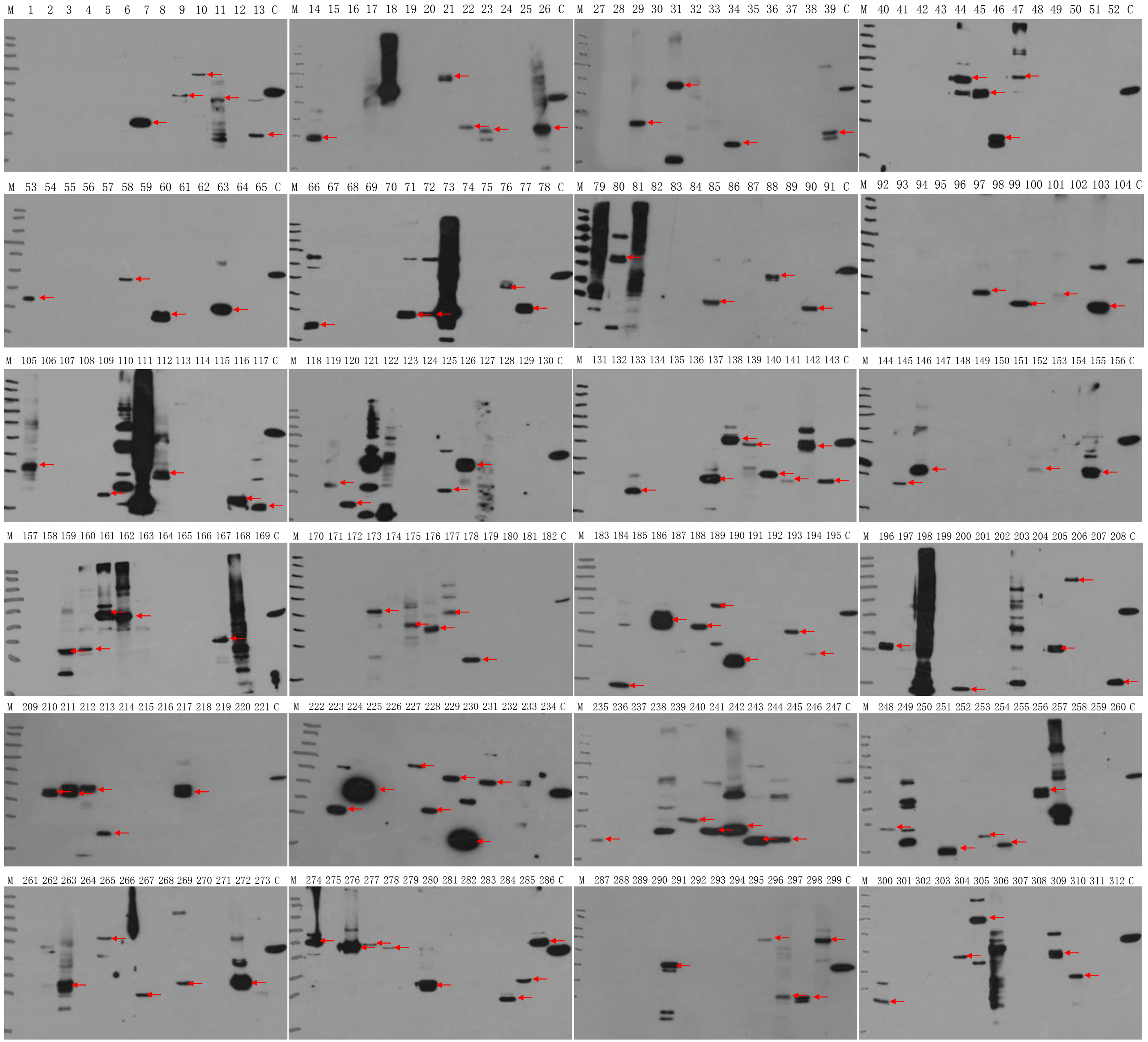

Supplemental Figure S2. Antibody quality was validated by western blot analyses with individual antibodies.

The validation of antibody quality was performed through Western blot analyses using individual antibodies. The process involved the separation of total protein using SDS-PAGE, followed by blotting onto PVDF (polyvinylidene fluoride) membranes. These membranes were then treated with the corresponding primary antibodies, succeeded by an HRP-conjugated anti-mouse IgG secondary antibody. Signal detection was carried out using a chemiluminescence system and exposure to X-ray film. The red arrow indicates the protein recognized by the antibody.

Supplemental Figure S3

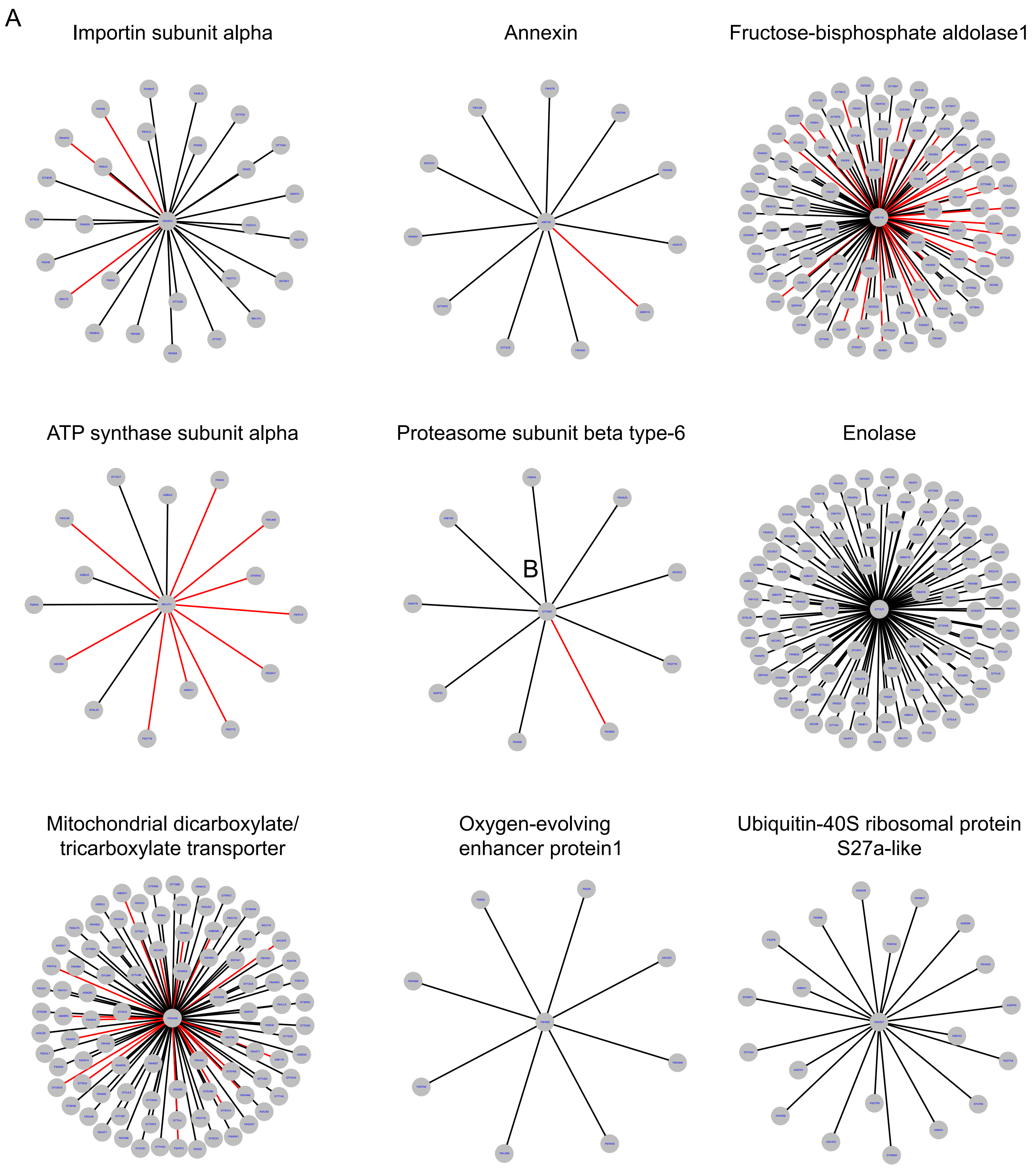

B

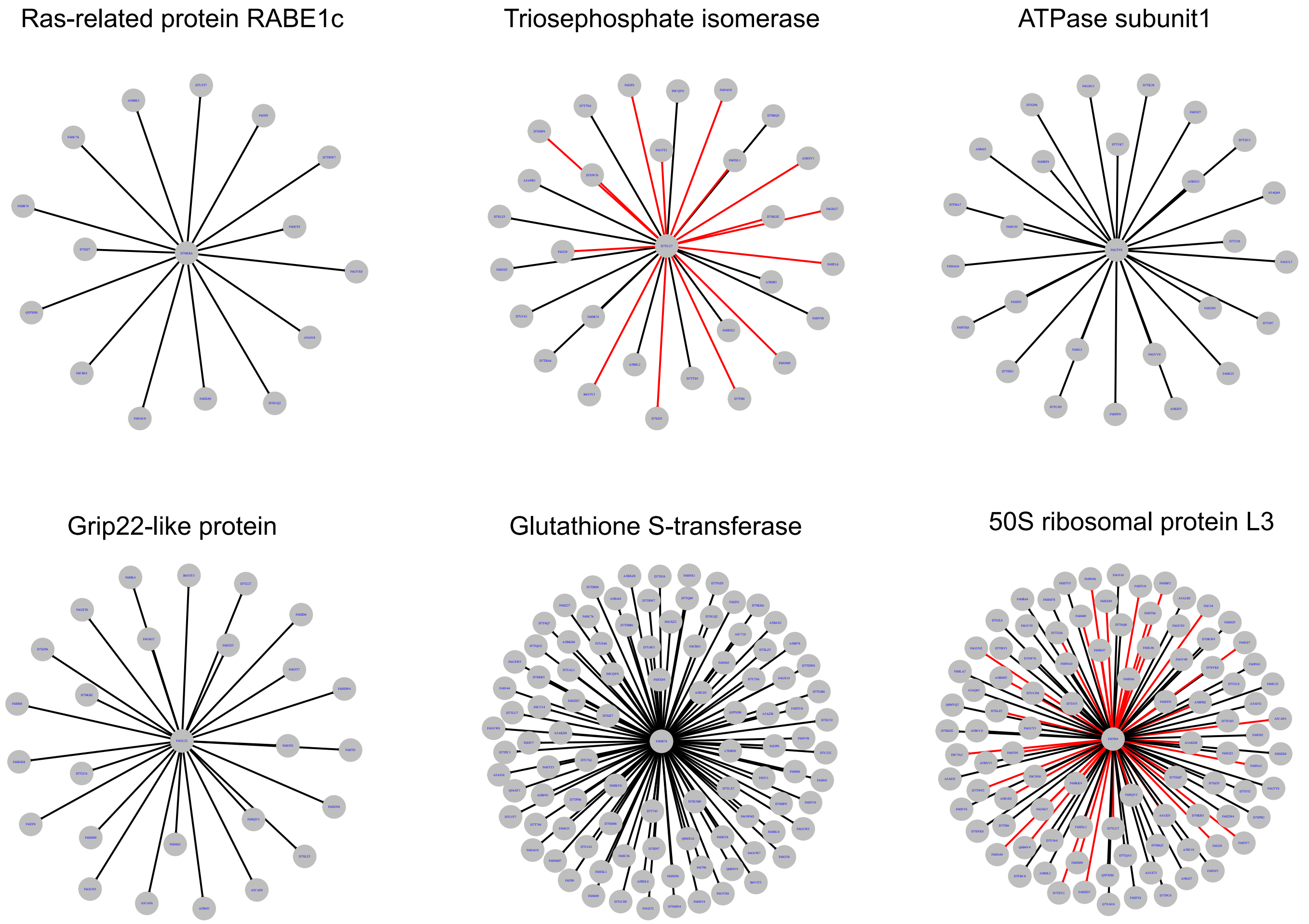
Ras-related protein RABE1c
Triosephosphate isomerase
ATPase subunit1
Grip22-like protein
Glutathione S-transferase
50S ribosomal protein L3

C

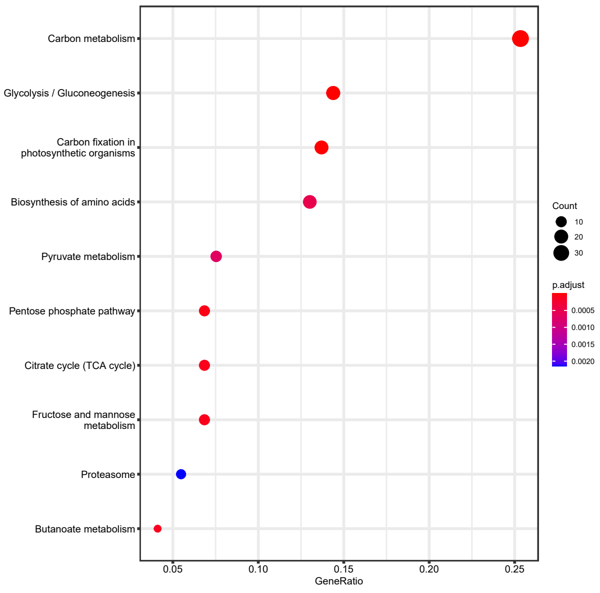
Carbon metabolism
Glycolysis / Gluconeogenesis
Carbon fixation in photosynthetic organisms
Biosynthesis of amino acids
Pyruvate metabolism
Pentose phosphate pathway
Citrate cycle (TCA cycle)
Fructose and mannose metabolism
Proteasome
Butanoate metabolism
0.05
0.10
0.15
0.20
0.25
GeneRatio
Count
10
20
30
p.adjust
0.0005
0.0010
0.0015
0.0020

D

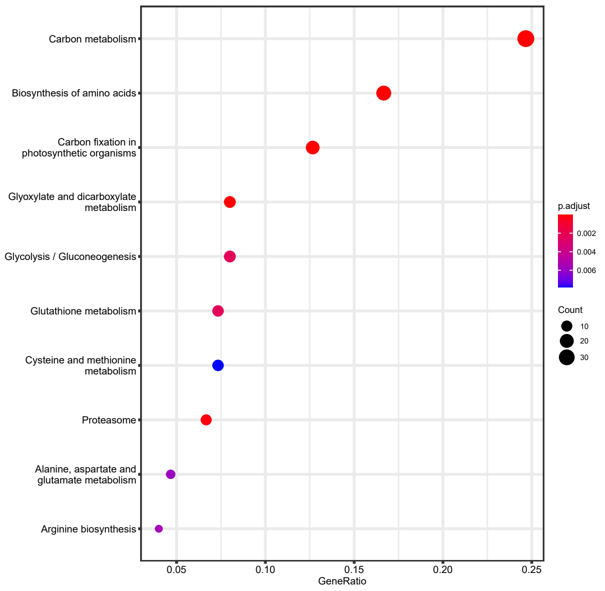
Carbon metabolism
Biosynthesis of amino acids
Carbon fixation in photosynthetic organisms
Glyoxylate and dicarboxylate metabolism
Glycolysis / Gluconeogenesis
Glutathione metabolism
Cysteine and methionine metabolism
Proteasome
Alanine, aspartate and glutamate metabolism
Arginine biosynthesis
0.05
0.10
0.15
0.20
0.25
GeneRatio
p.adjust
0.002
0.004
0.006
Count
10
20
30

Supplemental Figure S3. PPI network of differentially expressed proteins by IP-MS.

A-B showcases the PPI network of proteins that exhibit high accumulation in seeded (A) and seedless (B) grapes. In C, the KEGG analysis identified interacting proteins enriched in seeded (C) and seedless (D) grapes.

Supplemental Figure S4

A

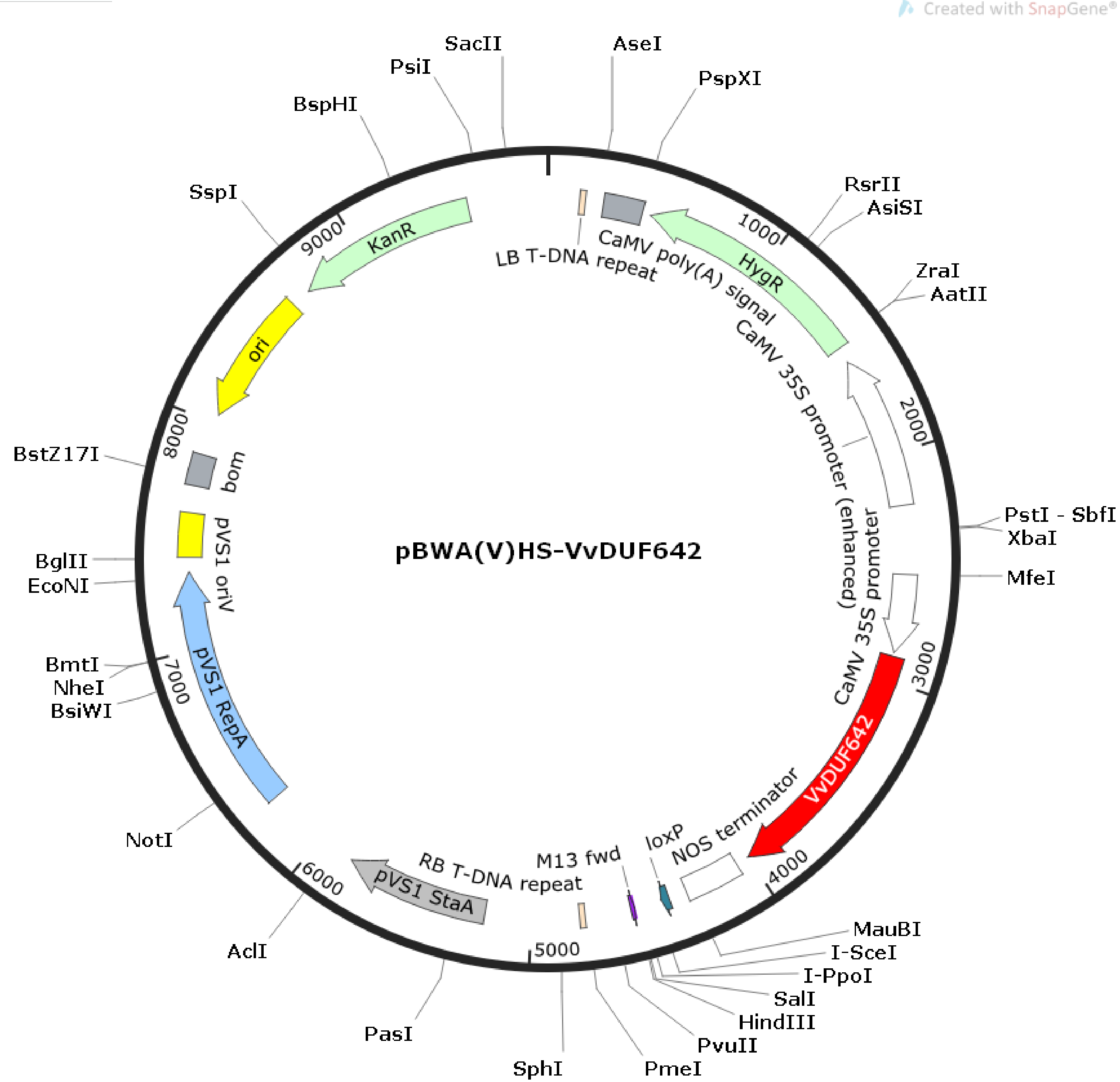

B

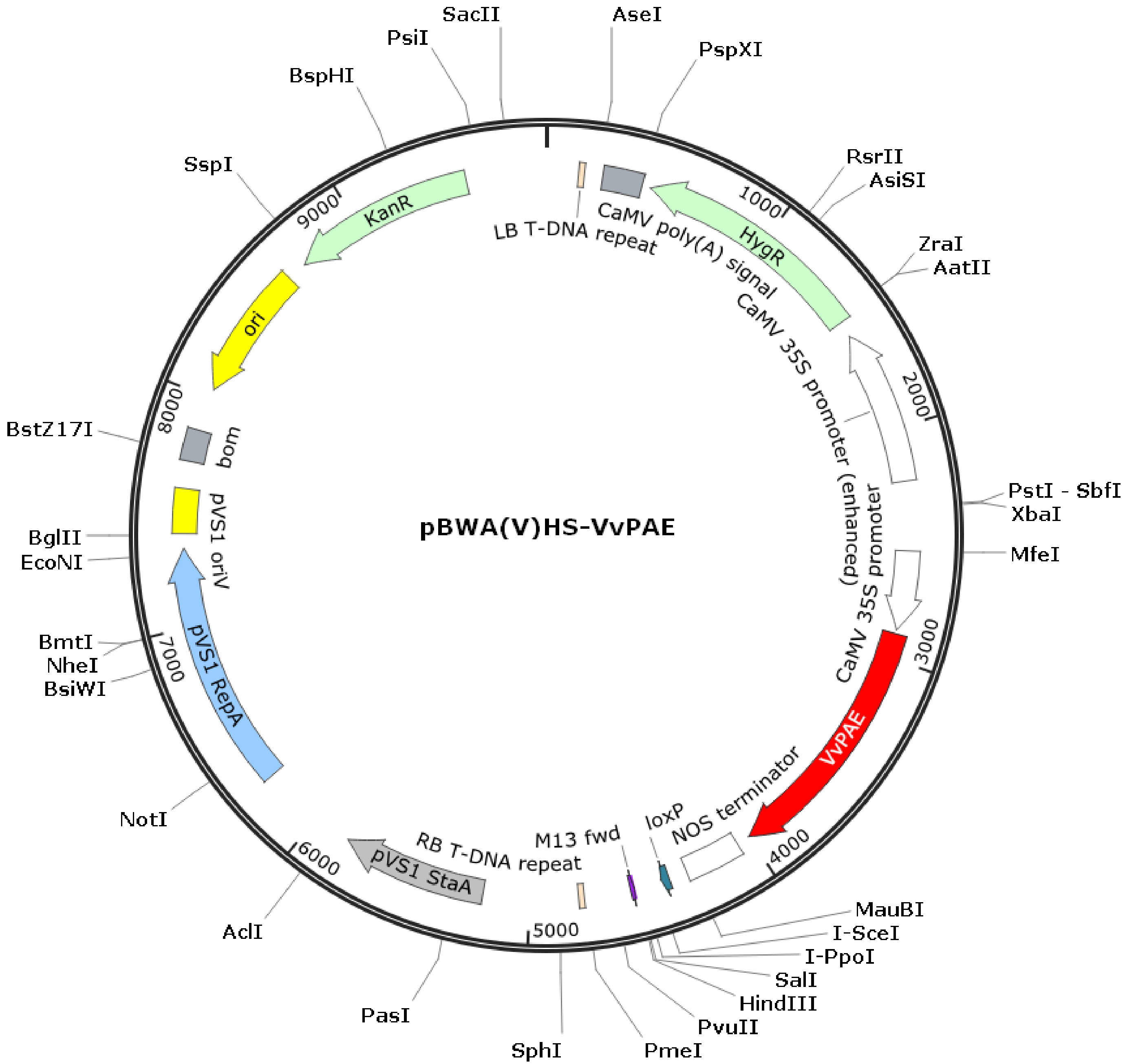

Created with SnapGene®
pBWA(V)HS-VvPAE
SacII
AseI
PsiI
PspXI
BspHI
SspI
RsrII
AsiSI
ZraI
AatII
PstI - SbfI
XbaI
MfeI
BstZ17I
BglII
EcoNI
BmtI
NheI
BsiWI
NotI
AclI
PasI
SphI
PmeI
PvuII
HindIII
SalI
I-PpoI
I-SceI
MauBI
KanR
ori
bom
pVS1 oriV
pVS1 RepA
pVS1 StaA
RB T-DNA repeat
M13 fwd
loxP
NOS terminator
VvPAE
CaMV 35S
CaMV 35S promoter (enhanced)
CaMV 35S promoter
HygR
CaMV poly(A) signal
LB T-DNA repeat
1000
2000
3000
4000
5000
6000
7000
8000
9000

Supplemental Figure S4. Tomato expression vector constructed. (A) *OE-VvDUF642* expression vector. (B) *OE-VvPAE* expression vector.
