# Supplementary material for: Antibody array-based proteome approach reveals proteins involved in grape seed development
Source: Plant Physiol. 2024 Feb 23;195(1):462–78. doi: 10.1093/plphys/kiad682 (PMC11060674; doi:10.1093/plphys/kiad682)
Supplement: kiad682_Supplementary_Data [file kiad682_supplementary_data.zip › PP2022RA01550R2_Supplemental_Tables.pdf]

Supplemental Data

Supplemental Table S1. Proteins identified by immunoprecipitation and mass spectrometry.

Antibody ID	UniProt No.	Description
V001927	A5BTZ8	Annexin
V002552	E0CQ27	COP9 signalosome complex subunit 4
V003286	D7T9G8	Assembly factor HCF136
V006462	F6HEJ3	Polyadenylate-binding protein RBP45
V007172	F6I0K4	Catalase
V007345	A5AHP0	Coatomer subunit beta
V007407	A5BEH3	Auxin-repressed protein
V007564	F6HZD0	ADP-ribosylation factor 2-like protein
V007772	D7TDX6	Photosystem I reaction center subunit II
V008608	F6HC36	Chalcone-flavonone isomerase family protein
V008767	A5ARC5	Importin subunit alpha
V008922	D7TKA5	Ornithine aminotransferase
V011709	F6I0M3	Diaminopimelate decarboxylase 2
V013091	F6GTY8	ATPase subunit 1
V013301	F6I581	Heat shock cognate protein 80
V013703	F6HG44	Glyceraldehyde-3-phosphate dehydrogenase
V014836	F6GXU1	40S ribosomal protein S4-3
V015037	F6HXC8	Phospholipase D
V015738	F6HZD7	Ubiquitin-40S ribosomal protein S27a-like
V018181	F6GTT2	ATP synthase subunit beta
V019714	D7TBH4	Malic enzyme
V019861	F6H5N5	Mitochondrial-processing peptidase subunit beta like
V021251	F6GTP8	Peptide methionine sulfoxide reductase
V024489	F6HR64	Putative 3-oxoacyl-[acyl-carrier-protein] reductase
V025649	F6HLL0	TBCC domain-containing protein 1
V025681	F6I523	Glutathione S-transferase
V026544	Q0ZIZ3	Photosystem II CP47 reaction center protein
V026615	F6HFF7	Phosphoglucomutase
V027471	F6H5F0	ADP, ATP carrier protein 3
V027698	D7TWP2	Carbonic anhydrase
V027963	D7TSJ8	Glutamine synthetase
V028925	D7TLU7	Triosephosphate isomerase
V029118	D7TFR9	F-actin-capping protein subunit beta
V029123	A5B1D3	Oxygen-evolving enhancer protein
V030323	C5DB51	Pyruvate kinase
V030446	F6H7H5	Bap31 domain-containing protein
V031354	F6HNX5	Heat shock cognate 70 kDa protein 2
V031772	E0CR49	Protein disulfide-isomerase

V031890	F6HHU0	2-Methylene-furan-3-one reductase
V032107	F6GTM7	Adenosylhomocysteinase
V032155	F6HNI6	Elongation factor 1-alpha
V032675	F6H3T7	DUF642
V033003	D7SKR5	L-ascorbate peroxidase 2
V033137	D7SS06	V-type proton ATPase catalytic subunit A
V033384	D7T0M7	Proteasome subunit beta type-6
V033452	F6I0H8	UTP--glucose-1-phosphate uridylyltransferase
V034407	A5ARE0	11S globulin subunit beta
V034491	A5C0T5	60S ribosomal protein L21-1
V034585	F6HMJ1	NAD(P)-bd_dom domain-containing protein
V034592	F6GUR4	Pyridoxal 5'-phosphate synthase subunit PDX1
V034602	F6GY46	Abscisic stress-ripening protein 2
V034778	A5BIN1	UDP-glucuronic acid decarboxylase 5
V035422	F6GY71	Pyruvate decarboxylase 2
V035539	D7TCD6	Aldehyde dehydrogenase family 7 member A1
V035959	B6VJY3	ATP synthase subunit alpha
V036511	F6HU55	Luminal-binding protein 5
V036764	D7TLX7	Ras-related protein RABE1c
V036800	F6HL98	Polygalacturonase inhibitor
V037366	F6GUN2	Tricarboxylate transporter DTC
V038279	F6HQV3	60S ribosomal protein L4
V039505	Q5PXH0	Aquaporin
V039941	F6I5H4	50S ribosomal protein L3
V042630	A5B118	Fructose-bisphosphate aldolase 1
V043659	F6GZ23	NAD(P)H dehydrogenase (quinone) FQR1-like
V044807	P56648	Ribulose bisphosphate carboxylase large chain
V049631	D7SIZ7	20 kDa chaperonin
V050403	A5BM68	TCTP domain-containing protein
V050590	F6GU22	Grip22-like protein
V050767	D7SYK8	ATP-citrate synthase beta chain protein 2
V052039	D7T227	Enolase
V053405	F6HX89	GTP-binding nuVear protein

Supplemental Table S2. List of proteins identified by VvDUF642 IP-MS.

UniProt No.	Unique Peptides	Description
F6H3T7	37	DUF642 GN=VIT_04s0008g05010
A5CAF6	26	Phosphoglycerate kinase GN=VIT_19s0085g00370
E0CQG0	17	Cellulase domain-containing protein GN=VIT_18s0001g12830
D7SKB2	11	ATPase_AAA_core domain-containing protein GN=VIT_06s0004g05180
F6GSG7	16	Glyceraldehyde-3-phosphate dehydrogenase

		GN=VIT_17s0000g10430
A5CAF8	24	Phosphoglycerate kinase GN=VIT_19s0085g00380
F6GSQ2	8	Glutamine synthetase GN=VIT_17s0000g01910
F6HG44	14	Glyceraldehyde-3-phosphate dehydrogenase GN=VIT_01s0010g02460
D7TQZ8	8	FMN hydroxy acid dehydrogenase domain-containing protein GN=VIT_19s0093g00510
D7SW04	18	Aspartate aminotransferase GN=VIT_07s0031g00980
A5B118	18	Fructose-bisphosphate aldolase GN=VIT_08s0007g03830
D7TWQ4	19	Formate dehydrogenase mitochondrial GN=VIT_14s0066g01320
A5AP38	7	Glutamine synthetase GN=VIT_01s0011g02200
D7TBL7	13	Aspartate aminotransferase GN=VIT_11s0016g03720
F6HBC6	11	Putative uncharacterized protein GN=VIT_13s0064g00460
D7FBC0	13	Malate dehydrogenase GN=VIT_07s0005g03360
F6GTE2	4	Alpha-1,4-glucan-protein synthase GN=VIT_17s0000g05030
D7TKA1	7	FMN hydroxy acid dehydrogenase domain-containing protein GN=VIT_10s0003g03830
F6H7Q2	14	Phosphoribulokinase GN=VIT_02s0109g00080
F6I0I5	2	Actin 7 GN=VIT_04s0044g00580
D7UDC9	12	Glyceraldehyde-3-phosphate dehydrogenase GN=VIT_18s0122g00960
F6I0K4	13	Catalase GN=VIT_04s0044g00020
F6HEZ8	3	Alpha-1,4-glucan-protein synthase GN=VIT_01s0011g00160
F6HA09	15	Aminotran_5 domain-containing protein GN=VIT_06s0009g03740
D7THJ7	3	ATPase_AAA_core domain-containing protein GN=VIT_08s0007g00840
D7U543	21	Glycerate dehydrogenase GN=VIT_03s0038g02510
D7U0Q3	16	Aminotran_1_2 domain-containing protein GN=VIT_09s0002g05250
F6H7I9	13	Aminomethyltransferase GN=VIT_10s0116g01540
D7TSJ8	2	Glutamine synthetase GN=VIT_14s0006g00350
F6HHQ7	12	Acetyl-CoA acetyltransferase, cytosolic 1 GN=VIT_12s0057g01200
Q0ZJ24	15	Photosystem II CP43 reaction center protein GN=psbC
F6HQV3	5	Ribos_L4_asso_C domain-containing protein GN=VIT_08s0040g03200
F6HNV2	2	ATPase_AAA_core domain-containing protein GN=VIT_13s0019g02050
P56648	14	Ribulose bisphosphate carboxylase large chain GN=rbcL
E0CP87	2	Patatin GN=VIT_18s0001g10830
F6H0C4	11	Phosphoprotein ECPP44 GN=VIT_18s0001g00360
F6HHU0	14	Pterin-binding domain-containing protein GN=VIT_01s0127g00740
F6GUN5	4	Ribos_L4_asso_C domain-containing protein GN=VIT_06s0004g00360
F6H409	10	Glyceraldehyde-3-phosphate dehydrogenase

		GN=VIT_14s0068g00680
F6HDW4	4	Epimerase domain-containing protein GN=VIT_05s0020g04510
F6HID6	15	Fatty acid hydroperoxide lyase 1 GN=VIT_12s0059g01060
A5ARE0	11	Glutelin type-A 1 GN=VIT_06s0004g04060
F6H5V8	9	At5g39570-like GN=VIT_14s0108g01160
D7TM77	13	PfkB domain-containing protein GN=VIT_13s0019g04470
F6HNF4	13	PfkB domain-containing protein GN=VIT_13s0019g04480
F6HZ64	11	Pectinesterase GN=VIT_07s0005g00730
Q0ZJ35	14	ATP synthase subunit alpha chloroplastic GN=atpA
D7TFE6	12	Pectin acetylesterase GN=VIT_02s0087g00400
Q0ZIZ3	12	Photosystem II CP47 reaction center protein GN=psbB
D7TJ46	16	Pyr_redox_2 domain-containing protein GN=VIT_08s0007g03610
Q0ZJ13	14	ATP synthase subunit beta chloroplastic GN=atpB
E0CRG5	13	Aminotran_1_2 domain-containing protein GN=VIT_18s0001g04860
F6HPJ1	3	Pyruvate dehydrogenase E1 component subunit alpha GN=VIT_01s0026g00990
D7TMH3	15	FBPase domain-containing protein GN=VIT_13s0019g03350
F6HM78	5	Malate dehydrogenase GN=VIT_10s0003g01000
A5B2Z7	6	Pyruvate dehydrogenase E1 component subunit alpha GN=VIT_09s0018g01940
D7T227	8	Phosphopyruvate hydratase GN=VIT_16s0022g01770
D7SUD7	4	Aspartate aminotransferase GN=VIT_04s0008g03770
D7T6P4	3	Glutamine synthetase GN=VIT_05s0020g02480
D7SYK8	10	ATP-citrate synthase GN=VIT_05s0077g00950
F6H3U9	11	Aspartate aminotransferase GN=VIT_04s0008g06040
D7TRJ7	7	Fumarylacetoacetase GN=VIT_00s0187g00340
F6HSN5	10	2-hydroxyacyl-CoA lyase GN=VIT_14s0006g02240
A5AIE0	10	Epimerase domain-containing protein GN=VIT_14s0060g00820
D7SHA5	10	PCI domain-containing protein GN=VIT_17s0000g10250
A5ASV7	14	GFO_IDH_MocA domain-containing protein GN=VIT_18s0001g01490
D7U6F9	10	Alpha-galactosidase GN=VIT_00s0375g00030
D7T2N7	9	Putative uncharacterized protein GN=VIT_05s0094g01520
F6HH42	9	Putative uncharacterized protein GN=VIT_11s0016g04290
F6HMN8	12	5-methyltetrahydropteroyltriglutamate--homocysteine methyltransferase GN=VIT_08s0056g01570
F6I4H0	2	S-adenosylmethionine synthase GN=VIT_14s0060g00480
F6HT17	10	PCI domain-containing protein GN=VIT_02s0012g02660
A5BEM6	10	Glutamate-1-semialdehyde 2,1-aminomutase GN=VIT_17s0000g00900
D7U3W2	7	CN hydrolase domain-containing protein GN=VIT_02s0033g00870
D7U3V8	7	CN hydrolase domain-containing protein GN=VIT_02s0033g00800
A5C0I8	3	Putative uncharacterized protein GN=VIT_04s0044g01110

D7T2G3	4	Putative uncharacterized protein GN=VIT_05s0094g00750
F6I7L9	5	Peptidase A1 domain-containing protein GN=VIT_00s1206g00010
F6HAM6	7	Transketolase GN=VIT_16s0022g01440
F6HXP6	8	40S ribosomal protein SA GN=VIT_09s0002g00610
F6HTI1	5	Fe2OG dioxygenase domain-containing protein GN=VIT_03s0017g00710
F6GVM7	7	Alpha-galactosidase GN=VIT_01s0137g00630
D7THV1	8	HP domain-containing protein GN=VIT_08s0007g08690
F6HL98	11	LRRNT_2 domain-containing protein GN=VIT_08s0007g07690
D7SLZ5	8	HABP4_PAI-RBP1 domain-containing protein GN=VIT_15s0021g01420
D7TQM9	8	Isocitrate dehydrogenase [NAD] subunit mitochondrial GN=VIT_08s0040g01700
F6I0H8	8	UTP--glucose-1-phosphate uridylyltransferase GN=VIT_04s0044g00710
B6VJY3	2	ATP synthase subunit alpha GN=atp1
D7UD99	6	Catalase GN=VIT_18s0122g01320
F6GZD1	3	PKS_ER domain-containing protein GN=VIT_00s0615g00030
D7TMY3	7	sorbitol dehydrogenase GN=VIT_16s0100g00300
E0CS20	6	PMR5N domain-containing protein GN=VIT_19s0014g00450
F6H8F3	6	Phosphoserine aminotransferase GN=VIT_00s2579g00010
D7TVV9	7	PKS_ER domain-containing protein GN=VIT_02s0025g03100
A2ICC9	6	Anthocyanidin synthase GN=ANS PE=2 SV=1
A5BIN1	5	NAD(P)-bd_dom domain-containing protein GN=VIT_13s0019g00840
D7TE48	2	AB hydrolase-1 domain-containing protein GN=VIT_12s0059g00230

Supplemental Table S3. Primers used in this study.

Name	Sequence	Usage
DUF642_For	CAGTGGTCTCACAACATGAGAGCTGTGGCGTTTCT	Gene Cloning
DUF642_Rev	CAGTGGTCTCATACATTAGATGTGCCTAGGAGGAG	
DUF642_Topo_For	CACCATGAGAGCTGTGGCGTTTCTTTTGC	BiFC, Co-IP
DUF642_Topo_Rev	GATGTGCCTAGGAGGAGTGCG	
VvPEC_Topo_For	ATGGTCGGGATTTCTATTATTTT	BiFC, Co-IP
VvPEC_Topo_Rev	AAGCTCATCTTTTGGGGGTGT	
VvPAE_Topo_For	CACCATGGCTAAAGCAAGAACAGGC	BiFC
VvPAE_Topo_Rev	TAATTCTGGGTGTTCGTGAGG	
DUF642_RT_For	AACTCTGTACAGCAGCAACG	RT-qPCR

DUF642_RT_Rev	AGCAACGGAATCGATCAACG	
VvPAE_RT_For	TGCAAGATTTCAGGGTGCAG	RT-qPCR
VvPAE_RT_Rev	TGCATTTCGGTTTGGCAGTG	
SlActin_For	TTTGCCGCATGCCATTCTTC	RT-qPCR
SlActin_Rev	TCATGTCGCGGACAATTTCC	
VvPAE_FLAG_For	AGAACACGGGGGACAAGCTTGGTACCATGGCTAAAGCAAG AACAGG	Transgenic plant constraction
VvPAE_FLAG_Rev	TCATGGTCTTTGTAGTCCATTAATTCTGGGTGTTCGTGAG	
SlGL3_For	ATACTCCGTCATCTGCGGGT	RT-qPCR
SlGL3_ Rev	ATCCTCGTCATCCTCAGCCT	
SICNR_For	TGGGAAGGGAAGAGAAGCATT	RT-qPCR
SICNR_ Rev	AAGAAGGATGTGCCGACCC	
SIETR1 _For	TGCTTGTCCACATCATTCCTG	RT-qPCR
SIETR1 _ Rev	TGTCTACCTGTCTCCTCCTGA	
SIARF7 _For	TTGAGTTCCAACATCGTGACC	RT-qPCR
SIARF7_ Rev	CACCAGTATCGCTTGCCCT	
SI AGL2_For	GGTCAGCAGCAACATCCTCA	RT-qPCR
SI AGL2_ Rev	ACAGCATCCAACCAGGTATCA	
SI AGL11_For	TGTGATGCTGAGATTGCTCT	RT-qPCR
SI AGL11_ Rev	CCCACCAGATGCCTATTTG	
SIAN1_For	GCTCCAAGTGTTACAAAGACA	RT-qPCR
SIAN1_ Rev	TAGGACCCACCTTAGCCTT	
